# Partial velocity slip effect on working magneto non-Newtonian nanofluids flow in solar collectors subject to change viscosity and thermal conductivity with temperature

**DOI:** 10.1371/journal.pone.0259881

**Published:** 2021-11-29

**Authors:** Wasim Jamshed, Mohamed R. Eid, Abederrahmane Aissa, Abed Mourad, Kottakkaran Sooppy Nisar, Faisal Shahzad, C. Ahamed Saleel, V. Vijayakumar

**Affiliations:** 1 Department of Mathematics, Capital University of Science and Technology (CUST), Islamabad, Pakistan; 2 Department of Mathematics, Faculty of Science, New Valley University, Al-Kharga, Al-Wadi Al-Gadid, Egypt; 3 Department of Mathematics, Faculty of Science, Northern Border University, Arar, Saudi Arabia; 4 Laboratoire de Physique Quantique de la Matière et Modélisation Mathématique (LPQ3M), University of Mascara, Mascara, Algeria; 5 Department of Mathematics, College of Arts and Sciences, Prince Sattam bin Abdulaziz University, Al-Kharj, Saudi Arabia; 6 Department of Mechanical Engineering, College of Engineering, King Khalid University, Asir-Abha, Saudi Arabia; 7 Department of Mathematics, School of Advanced Sciences, Vellore Institute of Technology, Vellore, Tamil Nadu, India; Central University of Karnataka, INDIA

## Abstract

Solar thermal collectors distribute, capture, and transform the solar energy into a solar thermal concentration device. The present paper provides a mathematical model for analyzing the flow characteristics and transport of heat to solar collectors (SCs) from non-Newtonian nanofluids. The non-Newtonian power-law scheme is considered for the nanofluid through partial slip constraints at the boundary of a porous flat surface. The nanofluid is assumed to differ in viscosity and thermal conductivity linearly with temperature changes and the magnetic field is appliqued to the stream in the transverse direction. The method of similarity conversion is used to convert the governing structure of partial differential formulas into the system of ordinary differential ones. Using the Keller box procedure, the outcoming ordinary differential formulas along with partial slip constraints are numerically resolved. A discussion on the flowing and heat transport characteristics of nanofluid influenced by power law index, Joule heating parameter, MHD parameter and slip parameters are included from a physical point of view. Comparison of temperature profiles showed a marked temperature increase in the boundary layer due to Joule heating. The thickness of the motion boundary-layer is minimized and the transport of heat through boundary-layer is improved with the partial slip velocity and magnetic parameters rising. Finally, With an increase in the Eckert number, the distribution of temperature within boundary layer is increased.

## 1. Introduction

Energy is an essential commodity that commands the functioning of our modern world. And as conventional, non-renewable sources of energy are being depleted faster than ever it’s crucial for us to look for alternative and renewable sources of energy. About 148×10^6^ km from the earth lies a star that emits so much energy that every hour 430×10^15^ kilojoules of its energy reach the earth that star is called the sun thus it’s abundantly clear that energy from the sun is more than enough to satisfy our energy demands. The question is how to collect solar energy.

Nowadays Harnessing the energy of the sun is done through two main methods photovoltaic and solar thermal collector the latter being preferable as it is cheaper and greatly more efficient Currently most of the research done on SCs is being concentrated on increasing their maximum thermal efficiency which is limited by the thermophysical of the absorber fluid. So when Choi [1] engineered the nanofluid and highlighted their exceptional properties its was no surprise that researchers started using them to boost the energy SCs competence.

The most popular solar panels for their prices and energy efficacy are flat solar panels and parabolic solar panels. Hence a great deal of research is done on the usage of nanoliquid as an inside liquid in these SCs. The thermal output of a solar flat plate (FPSC) collector loaded with water-TiO_2_ nanofluid was deliberated by Moravej et al. [2]. Using the ASHRAE standard, they found that the maximum thermal effectiveness of the FPSC is almost 78% which represents 9.80% gains in efficiency compared to the water baseline. In addition, increasing the concentration of particles yielded significant efficiency gains up to 33.54% at 5% nanoparticles concentration. Hussein et al. [3] employed a hybrid nanofluid where they recorded an 85% maximum thermal efficiency of the FPSC at 4 L/min flow, they also found that the increase in nanoparticle concentration improved the thermal energy increment and brought about a higher fluid outlet temperature. Saffarian et al. [4] investigated the influence of changing the flowing path of the nanofluid on FPSC thermal efficiency (U-shaped, wavy, and spiral). They showed that using CuO/water nanofluid and wavy pipes at a 4% wt. of solid fraction improved the heat transport rate up to 78.25%. Ziyadanogullari et al. [5] tested the thermal capability improvement of FPSC employing three water-based nanofluids (Al_2_O_3_, CuO, and TiO_2_) their results indicate that when compared with water, all of the tested nanofluids enhanced the collector efficiency with CuO-water nanofluid being the best one result in the large thermal conductance factor of its solid particles. These results agree with the findings of Allouhi et al. [6]. However, it’s important to note that when nanofluid is used, the efficiency is sensitive to changes in the operating conditions so it is essential to find the optimal working condition like solar radiation, size, and concentration of nanoparticles to get the best of what nanofluid can give, Tong et al. [7]. Moghadam et al. [8] developed an artificial neural network to optimize the turbulent flow of Al_2_O_3_ nanofluid inside a parabolic trough solar collector (PTSC). The results indicate that there is an optimal volume fraction for each average flow temperature and nanoparticle size. Subramani et al. [9] have considered the impact of CNT coating and using Al_2_O_3_ nanoliquid as occupying fluid on the efficiency of PTSC they reported that the combination of using nanofluid as working fluid and coating agent increased the maximum collector efficiency by 8.6% compared to regular PTSC using water. Khan et al. [10] compared the performances of a nanofluid inside a PTSC when the absorber tube geometry is varied. The combination of employing nanofluids and twisted tape insertion gave the best thermal performance. However, these thermal enhancement techniques have a major drawback as they produce a higher-pressure drop which renders the SC in need of higher pumping power. Moreover, The survey studies of Bellos et al. [11] and Kumar et al. [12] covered the best part of the literature on the use of nanofluids to improve PTSC thermal proficiency. Many other scientists have studied the use of nano-fluids as fluid works in a flat plate and they have all agreed on the positive influence the use of these common forms of solar collectors have on the competence of energy [13–17].

Although, the authors in the aforementioned studies mostly studied the convective flow of nanofluids using Newtonian fluid models. It is more realistic to use non-Newtonian fluid models on account of nanofluids exhibiting behavior as this is similar to that of non-Newtonian fluids. Some authors employed the non-Newtonian models in their studies of nanofluids’ thermal behavior under various thermophysical conditions, for example, Bahiraei et al. [18] debated the convective heat transportation of power law (TiO_2_) nanofluid and its entropy generation when it flowed inside a narrow annulus. Vahabzadeh et al [19] and Zhixiong et al. [20] employed the mixture model and considered the base fluid as non-Newtonian to simulate the mixed convective nanofluid flowing via a cavity containing warmer and coolant rotating cylinders. Aboud et al. [21] investigated the MHD influence on the mixed convective flow through a circular insertion occupied with power law nanofluid. Rehman et al. [22] calculated the entropy production result in thermophoretic and Brownian diffusion in non-Newtonian nanoliquid flow they also set the regular flux of solid particles at the expandable surface at zero. Raizah et al. [23] examined non-Newtonian nanofluid free convective flow in an oblique open shallow cavity saturated with permeable media using two-phase Buongiorno’s scheme. Asif et al. [24] applied the non-Newtonian Maxwell fluid model to study within a boundary layer the flow and temperature distribution of two water-based nanofluids (TiO_2_ and Cu) they scrutinized the effect of many parameters the qualitative analysis they carried out can be quantified to estimate the thermal effectiveness of SCs exploitation TiO_2_ or Cu-H_2_O nanofluid as a standard liquid. Mahmood et al. [25] conducted roughly the same study but they added analysis of entropy production in the structure. Outcomes depict that the entropy raises with the increment of many control parameters like Brinkmann number, Reynolds number, permeability parameter, and other ones. The nanofluid Cu-H_2_O has proved stronger in thermal conductance than TiO_2_-H_2_O nanofluid in these two investigations. Shooting methods have been employed by Jamshed and Aziz [26] for the numerical solution of nanofluid-driven power law MHD on moving surfaces of varying thermal conductivity. They also employed the Tiwari and Das nanofluid model and have detected that a larger magnetic field has a negative effect on the motion of the fluid particles inside the boundary layer.

Goyal et al. [27] investigated the 2D incompressible power-law nanoliquid via a stretchable plate under the effects of thermo-diffusive via Galerkin finite-element method (GFEM). They concluded that an augmentation in the value of “*Ln”* will decline the heat transport rate, while increase mass, nano, and regular rates of transportation. Finally, Usman et al. [28] examined the power-law non-Newtonian nanoliquid through a spinning disk occupied with gyrotactic micro-organisms. They used the shooting procedure to discuss the numerical outcomes and the effects of various physical parameters on the flow and heat transfer. In addition, it is noted that the emerging parameters have produced a major impact upon the velocity, energy, solid fraction profiles, and motile density of micro-organisms. For other studies regarding non-Newtonian nanofluid flows, one can consult [29–36].

The SCs are an indispensable component of the solar thermal apparatus and in order to build a good and efficient SC working with nanofluid, its design analysis must include entropy generation and exergy destruction computation. Alim et al. [37] and Tong et al. [38] performed a comparative examination of flat surface SCs operating with various nanoliquids from the viewpoint of entropy generation, energy efficiency, and exergy destruction. The findings suggest that the application of nano fluids increases exergy performance and decreases SC entropy production, and the magnitude of these effects depends significantly on the form and concentration of the nanoparticles in use. Alsarraf et al. [39] studied the impact of varying the shapes of nanoparticles on the rendering of MoS_2_/H_2_O nanoliquid inside SC. According to their results, blade-shaped nanoparticles produced the lowest level of entropy at *φ* = 4%, ṁ = 0.5 kg/s proving that the shape of nanoparticles also affects the entropy generation and energy competence of the structure. Eltaweel et al. [40] employed MWCNT/H_2_O nanoliquid in their exergetic and energetic examination of SC. They found that the increment of mass transport rate and the solid nanoparticles improves the exergy and energy efficiencies. Jamshed et al. [41] achieved a comparative examination of entropy generation in the thermal solar system using two different non-Newtonian Powell-Eyring nanofluids. The outcomes exhibit that The structure entropy upsurges whenever there is an increment in the values of one of these parameters: Brinkman, Reynolds numbers, radiative flow, surface convective parameters, nanoparticle concentration. They also observed that the spheres nanoparticle had the lowest rate of entropy generation.

The findings of variable viscosity, variable thermal conductivity, and the applied magnetic transverse field on partial slip-flow and heat transfer properties of non-Newtonian nanofluid power-law on porous flat surfaces were never analyzed in literature based on the above-mentioned survey of past literature and authors’ knowledge. In the present study, a mathematical flow model is offered to examine the flowing and heat transport features of a non-Newtonian nanofluid inside SCs and the influences of change in different overriding factors on velocity and energy outlines. The results are presented for velocity profiles the outline of temperature within the boundary-layer along with the drag force factor and number of Nusselt. The debate concludes with the characteristics of flow and heat transfer of nanofluid affected by different governing parameters within the boundary layer.

## 2. Problem statement and formulation

We consider a time-independent, laminar 2-D incompressible electrical conductive fluid of a power law nanofluid through a permeable surface. The surface acknowledges the partial slippage and is perceived as differing in linear temperature with the viscosity and temperature conductance of the nanofluid. The *y*-axis is normal to the permeable surface which lies on *x*-axis. A regular magnetic force *B*_*o*_ is employed normal to the surface and the induced magnetic field about the utilized magnetic domain is considered omittable. The surface temperature is *T*_*w*_ and the flow away from the platform is normal and parallel to the surface orientation. The ambient velocity and temperature afar from the plate is *u*_∞_ and *T*_∞_ respectively. Finally, there is a fixed injection (suction) velocity *V*_*w*_ crossways the plate.

**Fig 1:**
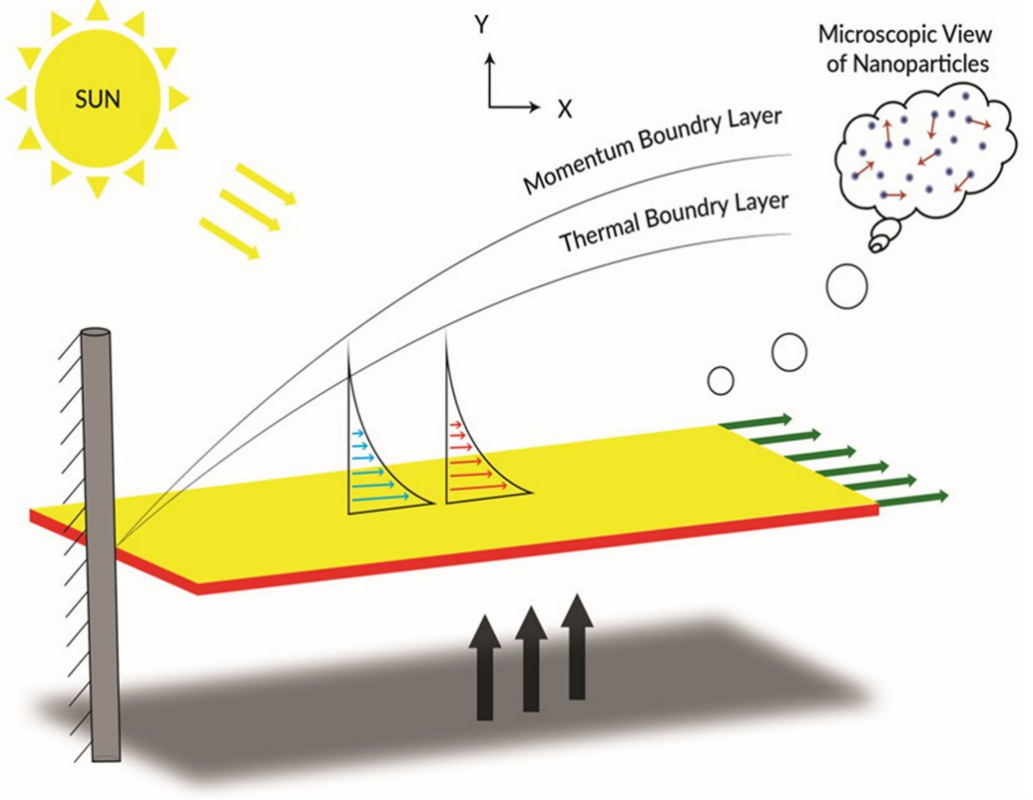
Diagram of the flow scheme.

The inwards structure of the PTSC is illustrated in Fig 1.

### 2.1. Model equations

The following method is based on the previous hypotheses and the normal boundary-layer approximation of PDEs (partial differential equations) for the flowing of power-law nanoliquid through heat transportation is gained as [42]

$$\frac{\partial u}{\partial x}+\frac{\partial v}{\partial y}=0,$$ (2.1)

$$u\frac{\partial u}{\partial x}+v\frac{\partial u}{\partial y}=\frac{1}{\rho_{nf}}\frac{\partial }{\partial y}\left[\mu_{nf}\left(T\right)|\frac{\partial u}{\partial y}{|}^{n-1}\frac{\partial u}{\partial y}\right]-\frac{\sigma_{nf}}{\rho_{nf}}B^{2}\left(u-U_{\infty }\right),$$ (2.2)

$$u\frac{\partial T}{\partial x}+v\frac{\partial T}{\partial y}=\frac{1}{{\left(\rho C_{p}\right)}_{nf}}\frac{\partial }{\partial y}\left[\kappa_{nf}\left(T\right)\frac{\partial T}{\partial y}\right]-\frac{1}{{\left(\rho C_{p}\right)}_{nf}}\frac{\partial q_{r}}{\partial y}+\frac{\sigma_{nf}}{{\left(\rho C_{p}\right)}_{nf}}B^{2}{\left(u-U_{\infty }\right)}^{2}.$$ (2.3)

Here *u* and *v* represent velocity in *x* and *y* orientations, correspondingly, *μ*_*nf*_(*T*) refers to dynamical viscosity, *ρ*_*nf*_ is the density, *σ*_*nf*_ is the electrical conductance, *T* is the temperature, (*C*_*p*_)_*nf*_ denotes the specific heat at a fixed pressure, *κ*_*nf*_(*T*) is the thermal conductance of the nanofluid and *q*_*r*_ is the flux of radiative heat.

### 2.2. Boundary equations

The required partial slip boundary-constraints for the suggested issue [42, 43]:

$$u=L_{1}{\left[\frac{\partial u}{\partial y}\right]}^{n},\;v=V_{w}\;at\;y=0;\;u\to u_{\infty }\;as\;y\to \infty ,$$ (2.4)

$$T=T_{w}+D_{1}\left[\frac{\partial T}{\partial y}\right]\;at\;y=0;\;T\to T_{\infty }\;as\;y\to \infty .$$ (2.5)

where $L_{1}=L\sqrt{R_{\text{e}}}$, $D_{1}=D\sqrt{R_{\text{e}}}$ are velocity and thermal slip factors with *L* and *D* are the preliminary amounts of swiftness and thermal slip coefficients.

### 2.3. Temperature dependent viscosity and thermal conductivity

The effect of the power law non-Newtonian nanofluid in the free convection heat transport through a porous layer to demonstrate temperature-dependent, efficient dynamic viscosity, and thermal conductivity, we considered [42, 44]

$$\mu_{nf}\left(T\right)=\mu_{nf}^{*}\left[a+b\left(T_{w}-T\right)\right],\;\;\kappa_{nf}\left(T\right)=\kappa_{nf}^{*}\left[1+\varepsilon \frac{T-T_{\infty }}{T_{w}-T_{\infty }}\right].$$ (2.6)

Here $\mu_{nf}^{*}$, $\kappa_{nf}^{*}$ are the constant value of the active dynamical viscosity and the thermal conductance respectively. Moreover, *a*, *b* and *ε* are the constant parameters with *b* > 0.

### 2.4. The thermophysical properties for power law nanofluids

Table 1 provides the thermophysical properties [45–47] for power nanofluids.

**Table 1:** Thermo-physical features for nanofluid.

Properties	Nanofluids
Dynamics viscosity	$\mu_{nf}=\mu_{f}{\left(1-\phi \right)}^{-2.5}$
Density	$\rho_{nf}=\left(1-\phi \right)\rho_{f}+\phi \rho_{s}$
Heat capacity	${\left(\rho C_{p}\right)}_{nf}=\left(1-\phi \right){\left(\rho C_{p}\right)}_{f}+\phi {\left(\rho C_{p}\right)}_{s}$
Thermal conductivity	$\frac{k_{nf}}{k_{f}}=\left[\frac{\left(k_{s}+\left(m-1\right)k_{f}\right)-\left(m-1\right)\phi \left(k_{f}-k_{s}\right)}{\left(k_{s}+\left(m-1\right)k_{f}\right)+\phi \left(k_{f}-k_{s}\right)}\right]$
Electrical Conductivity	$\frac{\sigma_{nf}}{\sigma_{f}}=\left[1+\frac{3\left(\frac{\sigma_{s}}{\sigma_{f}}-1\right)\phi }{\left(\frac{\sigma_{s}}{\sigma_{f}}+2\right)-\left(\frac{\sigma_{s}}{\sigma_{f}}-1\right)\phi }\right]$

In Table 1, *ϕ* is solid fraction factor *μ*_*f*_, *ρ*_*f*_ and (*C*_*p*_)_*f*_ are the fluid dynamical viscosity, density, and specific heat, whilst *μ*_*f*_, *ρ*_*s*_ and (*C*_*p*_)_*s*_ are the same characteristics for solid nanoparticles, *κ*_*nf*_ is thermal conductance, *κ*_*f*_, *σ*_*f*_, and *κ*_*s*_, σ_*s*_ signify the thermal and electrical conductances for liquid and solid particles.

### 2.5. Rosseland approximation

The flux of radiative heat is calculated by (see for details, Brewster [48]):

$$\frac{\partial q_{r}}{\partial y}=-\frac{16T_{\infty }^{3}\sigma^{*}}{3k^{*}}\frac{\partial^{2}T}{\partial y^{2}}.$$ (2.7)

In Eq (2.7), k* is the coefficient of mean absorption and σ* is the constant value of Stefan Boltzmann.

## 3. Problem solve

This investigation uses a technique of similarities to solve formulas system (2.1)–(2.3) along with the boundary constraints (2.4)–(2.5). The stream function *ψ*(*x*,*y*) is familiarized which exactly validates Eq (2.1) with

$$u=\frac{\partial \psi }{\partial y},\;v=-\frac{\partial \psi }{\partial x}.$$ (3.1)

Eq (2.2) after using Eqs (2.6) and (3.1) is transformed into

$$\begin{array}{l} \frac{\partial \psi }{\partial y}\frac{\partial^{2}\psi }{\partial x\partial y}-\frac{\partial \psi }{\partial x}\frac{\partial^{2}\psi }{\partial y^{2}}=\frac{\mu_{nf}^{*}}{\rho_{nf}}\left[-b\frac{\partial T}{\partial y}|\frac{\partial^{2}\psi }{\partial y^{2}}{|}^{n-1}\frac{\partial^{2}\psi }{\partial y^{2}}\right]-\frac{\sigma_{nf}}{\rho_{nf}}B^{2}\left(\frac{\partial \psi }{\partial y}-u_{\infty }\right)\\ \;+\frac{\mu_{nf}^{*}}{\rho_{nf}}\left[\left[a+b\left(T_{w}-T\right)\right]\{\left(n-1\right)|\frac{\partial^{2}\psi }{\partial y^{2}}{|}^{n-1}{\left(\frac{\partial^{2}\psi }{\partial y^{2}}\right)}^{2}\frac{\partial^{3}\psi }{\partial y^{3}}+|\frac{\partial^{2}\psi }{\partial y^{2}}{|}^{n-1}\frac{\partial^{3}\psi }{\partial y^{3}}\}\right] \end{array}.$$ (3.2)

Similarly Eq (2.3) after using Eqs (2.7)–(3.1) is transformed into

$$\begin{array}{ll} \frac{\partial \psi }{\partial y}\frac{\partial T}{\partial x}-\frac{\partial \psi }{\partial x}\frac{\partial T}{\partial y}= & \frac{\kappa_{nf}^{*}}{{\left(\rho C_{p}\right)}_{nf}}\left[\frac{\epsilon }{T_{w}-T_{\infty }}\right]{\left(\frac{\partial T}{\partial y}\right)}^{2}+\frac{\kappa_{nf}^{*}}{{\left(\rho C_{p}\right)}_{nf}}\left[1+\epsilon \frac{T-T_{\infty }}{T_{w}-T_{\infty }}\right]\frac{\partial^{2}T}{\partial y^{2}}\\ & +\frac{16}{3k^{*}}T_{\infty }^{3}\sigma^{*}\frac{\partial^{2}T}{\partial y^{2}}\frac{1}{{\left(\rho C_{p}\right)}_{nf}}+\frac{\sigma_{nf}}{{\left(\rho C_{p}\right)}_{nf}}B^{2}\left(\frac{\partial \psi }{\partial y}-u_{\infty }\right) \end{array}.$$ (3.3)

Boundary conditions (2.4) likewise transformed into

$$\frac{\partial \psi }{\partial y}=L_{1}\frac{\partial^{2}\psi }{\partial y^{2}}{\left|\frac{\partial^{2}\psi }{\partial y^{2}}\right|}^{n-1},\;\frac{\partial \psi }{\partial x}=-V_{w},\;at\;y=0;\;\frac{\partial \psi }{\partial y}\to 0\;as\;y\to \infty .$$ (3.4)

In order to get the dimensionless form of the scheme of PDE’s (3.2)–(3.3) we announce the non-dimensional similarity conversion

$$\eta ={\left(\frac{R_{e}}{x/L}\right)}^{\frac{1}{n+1}}\frac{y}{L}.$$ (3.5)

The non-dimensional function of stream *f*(*η*) and non-dimensional temperature *θ*(*η*) are

$$\psi \left(x,y\right)=Lu_{\infty }{\left(\frac{x/L}{R_{e}}\right)}^{\frac{1}{n+1}}f\left(\eta \right),\;\theta \left(\eta \right)=\frac{T-T_{\infty }}{T_{w}-T_{\infty }},$$ (3.6)

Introducing the above transformations in Eqs (3.2) and (3.3), we obtain the self similar set of ODE’s

$$n\left(a+A-A\theta \right){\left|f\prime \prime \right|}^{n-1}f\prime \prime \prime +\left(\frac{\phi_{2}}{\phi_{1}}\right)\left(\frac{1}{n+1}\right)ff\prime \prime -A\theta \prime {\left|f\prime \prime \right|}^{n}-\left(\phi_{4}/\phi_{1}\right)M\left(f'-1\right)=0,$$ (3.7)

$$\left(1+\varepsilon \theta +\frac{4}{3}Rd\right)\theta ''+\varepsilon {\theta \prime }^{2}+\frac{\phi_{3}}{\phi_{5}}\frac{f\theta 'Pr}{n+1}+\frac{\phi_{4}}{\phi_{5}}MEcPr{\left(f'-1\right)}^{2}=0.$$ (3.8)

Where, $M=\frac{\sigma_{f}B^{2}x}{\rho_{f}u_{\infty }}$ is a megnetic parameter, *A* = *b*(*T*_*w*_—*T*_∞_) is the viscosity parameter, $Pr={\left(\frac{u_{\infty }^{3}}{x}\right)}^{\frac{n-1}{n+1}}\left(\frac{C_{pf}}{kf}\right){\left(\frac{\mu_{f}}{\rho_{f}}\right)}^{\frac{2}{n+1}}$ is the Prandtl number, $Ec=\frac{u_{\infty }^{2}}{{\left(C_{p}\right)}_{f}\left(T_{w}-T_{\infty }\right)}$ and $R_{e}=\frac{\rho_{f}L^{n}}{\mu_{f}u_{\infty }^{\left(n-2\right)}}$ is the number of Reynold.

where,

$$\phi_{1}={\left(1-\phi \right)}^{2.5},\;\phi_{2}=\left(1-\phi +\phi \frac{\rho_{s}}{\rho_{f}}\right),\;\phi_{3}=\left(1-\phi +\phi \frac{{\left(\rho C_{p}\right)}_{s}}{{\left(\rho C_{p}\right)}_{f}}\right),$$ (3.9)

$$\phi_{4}=\left(1+\frac{3\left(\frac{\sigma_{s}}{\sigma_{f}}-1\right)\phi }{\left(\frac{\sigma_{s}}{\sigma_{f}}+2\right)-\left(\frac{\sigma_{s}}{\sigma_{f}}-1\right)\phi }\right),\;\phi_{5}=\left(\frac{\left(k_{s}+2k_{f}\right)-2\phi \left(k_{f}-k_{s}\right)}{\left(k_{s}+2k_{f}\right)+\phi \left(k_{f}-k_{s}\right)}\right).$$ (3.10)

The boundary constraints (3.4) and (2.5) are transformed to the next form:

$$f\left(\eta \right)=S,\;f'\left(\eta \right)=\delta f''\left(\eta \right){\left|f''\left(\eta \right)\right|}^{n-1}\;at\;\eta =0;\;f'\left(\eta \right)\to 1\;as\;\eta \to \infty ,$$ (3.11)

θ(η)=1+Δθ'(η)atη=0;θ(η)→0asη→∞, (3.12)

where $S=-\frac{\left(n+1\right)x^{\frac{n}{n+1}}}{{\left(u_{\infty }\right)}^{\frac{2n-1}{n+1}}}{\left(\frac{\rho_{f}}{\mu_{f}}\right)}^{\frac{1}{n+1}}V_{w}$ is the injection (suction) agrees with suction at what time *S* > 0 and corresponds to injection when *S* < 0. $\delta =\frac{L^{\frac{n+2}{2}}}{{\left(u_{\infty }\right)}^{\frac{n\left(n-5\right)}{2n+2}}}{\left(\frac{\rho_{f}}{\mu_{f}}\right)}^{\frac{3n+1}{2n+2}}$ is the velocity slip parameter and $\Delta =D{\left(\frac{L^{n\left(n+2\right)}}{x}\right)}^{\frac{1}{n+1}}{\left(\frac{\rho_{f}}{\mu_{f}}\right)}^{\frac{n+3}{n+1}}$ is the parameter of thermal slip.

## 4. Computational procedure

**Fig 2:**
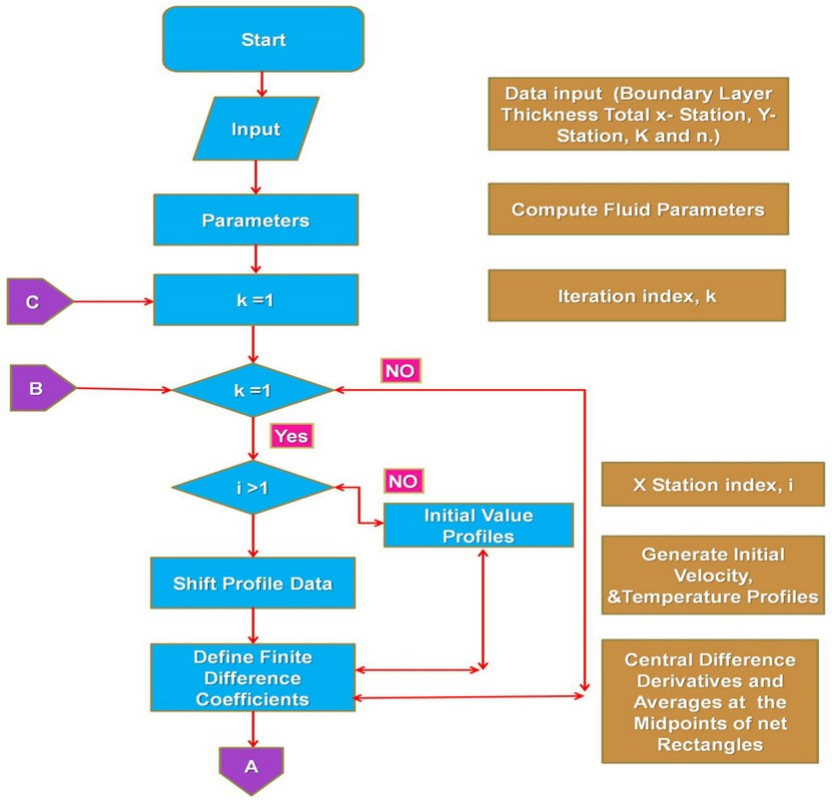
Keller box technique flow chart (continue).

**Fig 3:**
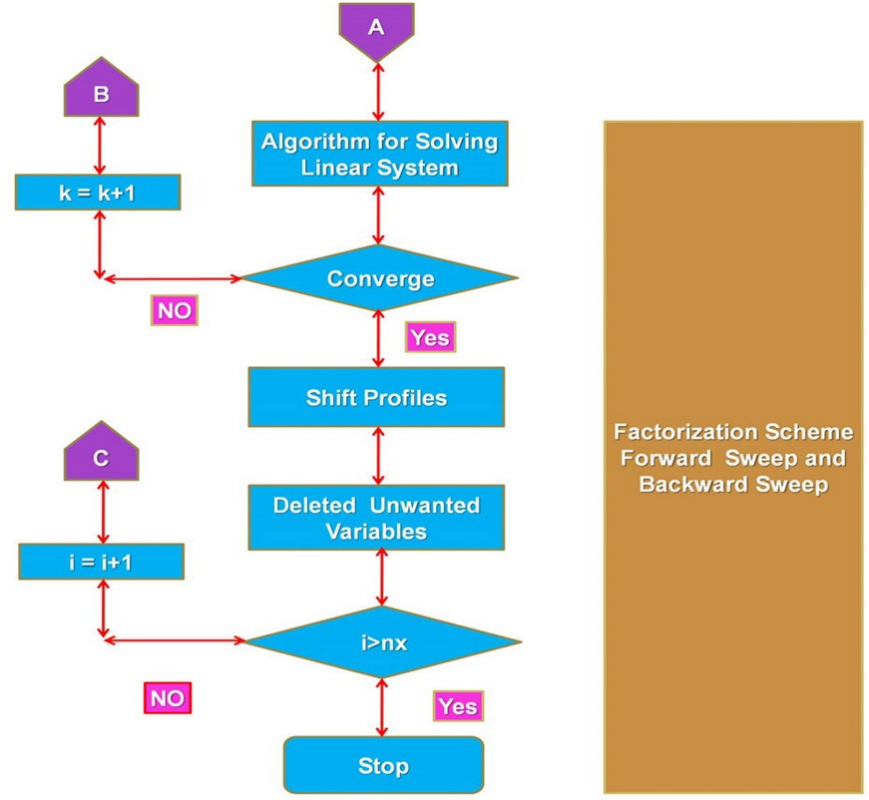
Keller box technique flow chart.

The set of nonlinear ODE’s (3.7)—(3.8), resulting from nanofluid flow mathematical modelling, is strenuous analytically to overcome. Therefore, a classical computing method called the Keller box [49] is used to evaluate the approximate outcomes. This approach is typically frequently used in the study of laminar BL flow. This system is fundamentally robust and convergent in a second order. In the following flow charts shown as Figs 2 and 3, the technique of the Keller box system is explained.

Initial strand of the method suggests transforming the ODEs (3.3)-(3.4) into odes of the first order.

$$f^{\prime }=p,$$ (4.1)

$$p^{\prime }=q,$$ (4.2)

θ'=z, (4.3)

$$n\left(a+A-A\theta \right){\left|q\right|}^{n-1}q^{\prime }+\left(\frac{\phi_{2}}{\phi_{1}}\right)\left(\frac{1}{n+1}\right)fq-Az|q{|}^{n}-\left(\phi_{4}/\phi_{1}\right)M\left(p-1\right)=0,$$ (4.4)

$$\left(1+\epsilon \theta +\frac{4}{3}Rd\right)z'+\epsilon z^{2}+\frac{\phi_{3}}{\phi_{5}}\frac{fzPr}{n+1}+\frac{\phi_{4}}{\phi_{5}}MEcPr{\left(p-1\right)}^{2}=0.$$ (4.5)

Presence of newly variables, boundary conditions eventually turn:

$$f\left(0\right)=S,\;p\left(0\right)=\delta q\left(0\right){\left|q\left(0\right)\right|}^{n-1},\;\theta \left(0\right)=1+\Delta z\left(0\right)\;\text{p}\left(\infty \right)\to 0,\theta \left(\infty \right)\to 0.$$ (4.6)

### 4.1. Code validation

For short, there are no specifics of the solution process. The findings are compared to the results available in the literature in order to validate the numerical method using the suggested code. The test status is MHD free convective of the boundary-layer flow of power-law fluid past a flat surface with Newtonian slip. The findings gained by this code are compared with the outcomes gotten in Table 2 by other researchers. These outcomes have been attained for *a* = 1, *ϕ* = *A* = *ε* = *S* = *Rd* = *Ec* = 0. The results calculated and those presented by Ref. [50] are outstanding accord as can be revealed in Table 2 for the coefficient of drag force and the number of Nusselt.

**Table 2:** Comparison values of *f*″(0) and –*θ′*(0).

*n*	*M*	*P*_*r*_	δ	Δ	Ref. [50] *f*″(0)	Ref. [50] –*θ*′(0)	Present *f*″(0)	Present –*θ*′(0)
0.4	0.6	0.7	0.3	0.3	0.82269	0.39319	0.822690	0.393190
1.0	0.6	0.7	0.3	0.3	0.68047	0.33497	0.680471	0.334972
1.4	0.6	0.7	0.3	0.3	0.67638	0.31484	0.676380	0.314843
1.4	0.6	0.7	0.3	0.0	0.50701	0.31484	0.50701	0.347683
1.4	0.6	0.7	0.3	0.0	0.67638	0.28767	0.676380	0.287672
1.4	1.0	0.7	0.3	0.3	0.78586	0.32410	0.785860	0.324101

## 5. Results and discussion

Numerical computations have been performed to investigate the effects of the parameters: power law *n*, viscosity *A*, thermal conductivity *ε*, solid concentration *ϕ*, magnetic parameter *M*, slip velocity *σ* suction/injection *S* and thermal slip Δ on velocity *f*′(*η*) and temperature *θ*(*η*) outlines of *Cu*–H_2_O nano-fluid. The numeric results for differences in *f*′(*η*) and *θ*(*η*) distributions are displayed in the form of graphs to show the useful relationship between the parameters. Also computed by the differences in the control parameters and tabulated under Table 4 is the surface drag force factor and heat transport rate in the soil.

The behavior of skin friction coefficient $C_{f}Re_{x}^{1/2}=\left(\frac{{\left(f''\left(0\right)\right)}^{n}}{{\left(1-\phi \right)}^{2.5}}\right)$ and Nusselt number $Nu_{x}\;Re_{x}^{-1/2}=-\frac{k_{nf}}{k_{f}}\left(1+\frac{4}{3}Rd\right)\theta '\left(0\right)$ (see for details, Hussian et al. [31]) behavior has been looked at in Table 4.

### 5.1. Nanofluid thermal characteristics

The standard fluid (H_2_O) and solid nanoparticle (Cu) thermo-physical features are specified in Table 3 [51].

**Table 3:** Thermo-physical features at 293 K.

Thermophysical Properties	*ρ*(*kg*/*m*^3^)	*C*_*p*_(*J*/*kg*.*K*)	*K*(*W*/*m*.*K*)	*σ*(*S*/*m*)
Water (H_2_O)	997.1	4179	0.6130	0.5×10^−6^
Copper (Cu)	8933	385	401	5.96×10^7^

### 5.2. Effect of viscosity parameter *A*

**Fig 4:**
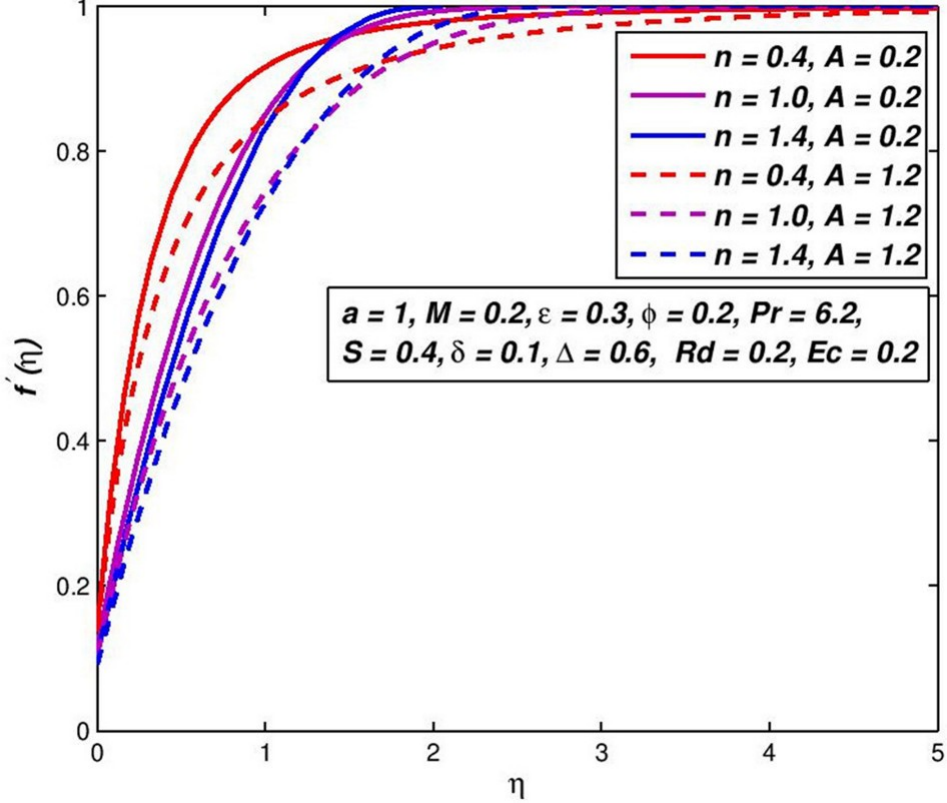
Effect of *A* on *f*′(*η*) for diverse *n* values.

**Fig 5:**
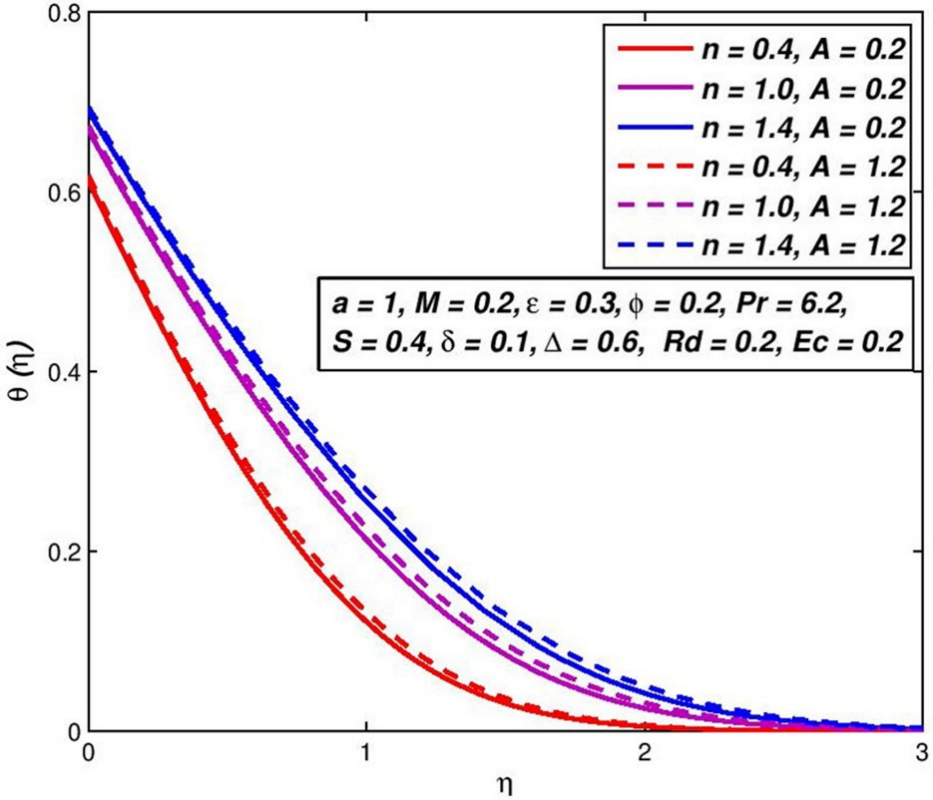
Effect of *A* on *θ*(*η*) for diverse *n* values.

The viscosity parameter *A* depends on the viscosity or the temperature features of the standard fluid and the working temperature change. The influence of variable viscosity on *f*′(*η*) and *θ*(*η*) outlines of Newtonian (*n* = 1), pseudoplastic (*n* < 1) and dilatant (*n* > 1) fluids are presented in Figs 4 and 5. The assessment of behaviors in Fig 4 shows the velocity profiles of power-law nanofluid upsurges in some initial range of *η* where the effect of viscosity is insignificant and decreases after that. The decreasing trend in velocity profiles results in the growth of resisting in fluid, produced by snowballing the viscosity value. In addition, results of *n* on non-Newtonian nanofluid velocity distribution may be observed from the fixed value parameter of the viscosity (say, *A* = 0.6) (Fig 4). In some initial range of *η*, the velocity of pseudoplastic liquids upsurges speedy, tracked by the Newtonian fluids and then the shear thickening fluids. This happened because there is the lowest effective viscosity in this range of shear diluent fluids. Under the dilating nanofluids, and subsequently the Newtonian nanofluid, the velocity of pseudoplastic nanofluids first falls. This opposite pattern reduces shear stress and the increased viscosity of the thinner liquids. The combined influences on the temperature outline of *n* and *A* as seen in Fig 5. The impact of increment values of *A*, result in temperature boundary-layer is more thickness, which consequences in an upsurge of energy and reduction in the rate of heat transportation. Compared to Newtonian and shear thinning liquids, this conduct is very evident in shear thickening liquids.

### 5.3. Effect of thermal conductivity parameter *ε*

**Fig 6:**
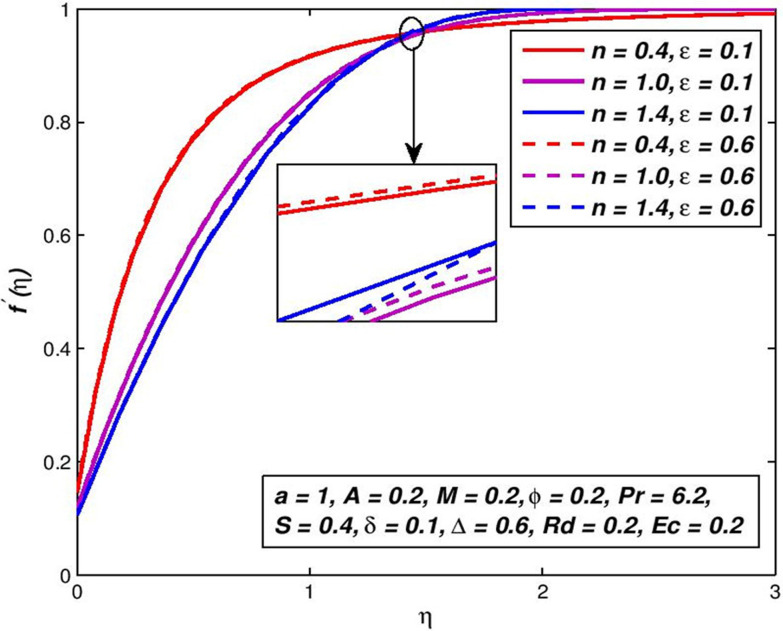
Effect of *ε* on *f*′(*η*) for diverse *n* values.

**Fig 7:**
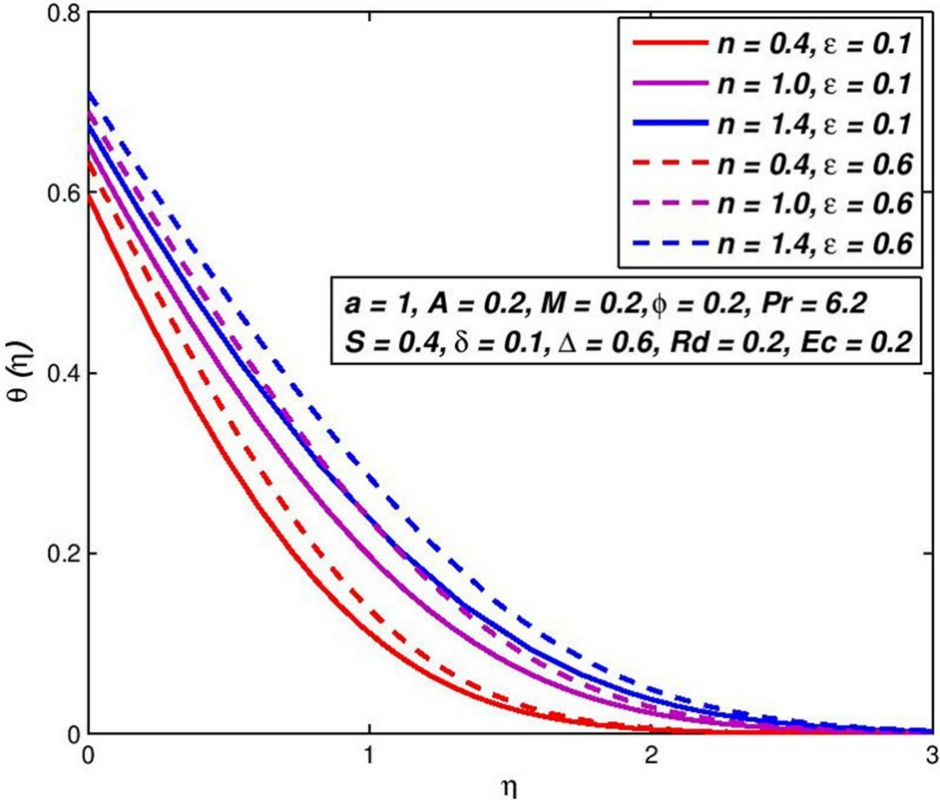
Effect of *ε* on *θ*(*η*) for diverse *n* values.

Figs 6 and 7 depict *f*′(*η*) and *θ*(*η*) outlines of non-Newtonian nanoliquid for change in thermal conductivity parameter *ε*. It is clear from Fig 6, there is no effect of parameter *ε* on *f*′(*η*) outlines of nanoliquid. Nevertheless, the snowballing amounts of *n*, shows the velocity boundary-layer thickening in about the primary range of *η* and after this, the velocity boundary-layer thinning is remarked. In the debate on Fig 4, the cause for this comportment is explained. Graph analyzes in Fig 7, it has been shown that the increase in thermal conductivity parameter *ε* is an upsurge in nanofluid temperature and tendencies to zero asymptotically as the distance from border upsurges. Our definition, $\kappa_{nf}>\kappa_{nf}^{*}$, shows this fact, that for *ε* > 0, i.e. nanofluid thermal conductivity increases with the swelling parameter value *ε*. Hence, lift the temperature from the flat surface for a certain distance. Thermal conductivity is a heat transfer measurement across one particular material. The thermal conductivity of refractories depends on temperature and generally is greater at high temperatures. This means that there is a positive relationship.

### 5.4. Effect of magnetic parameter *M*

**Fig 8:**
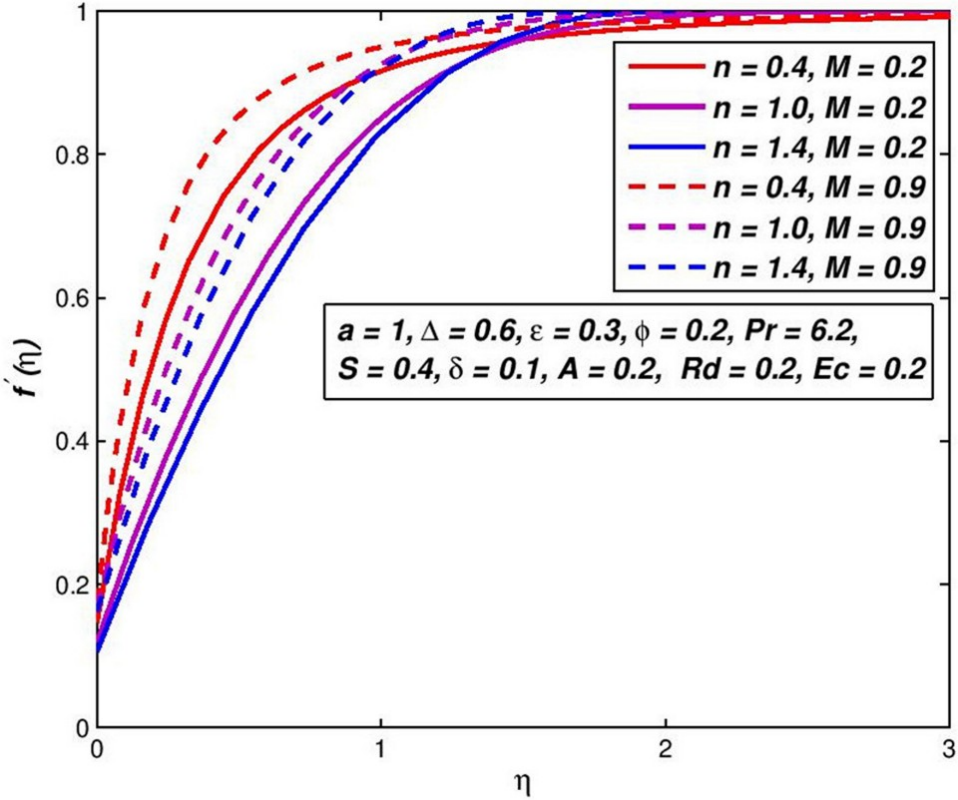
Effect of *M* on *f*′(*η*) for diverse *n* values.

**Fig 9:**
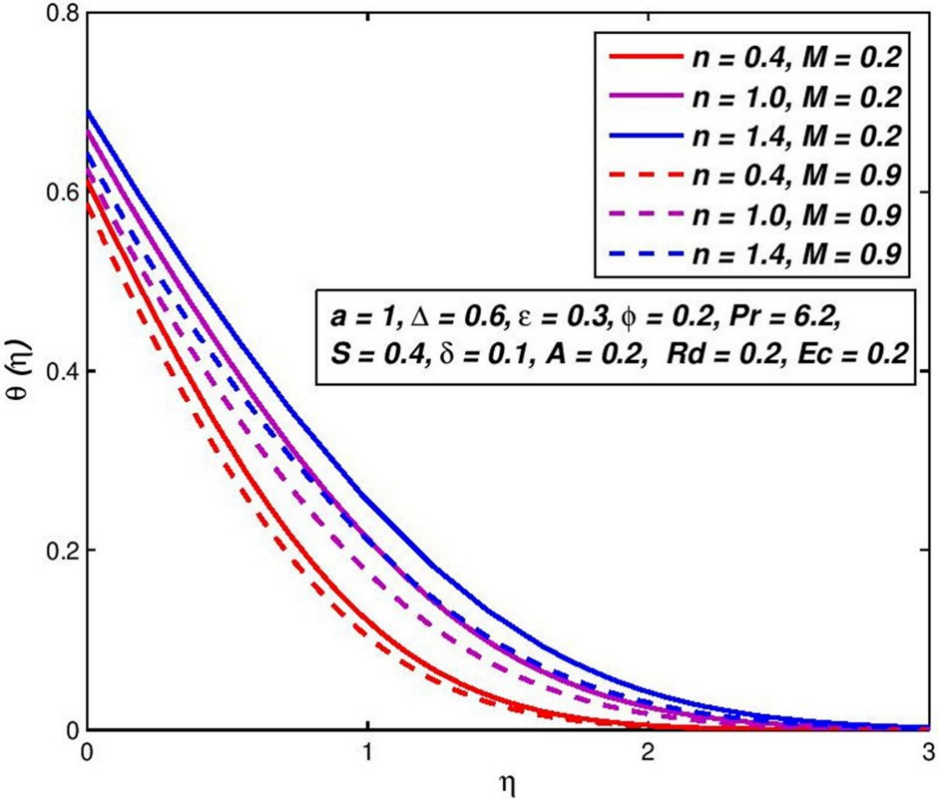
Effect of *M* on *θ*(*η*) for diverse *n* values.

The consequence of the difference in the fluid velocity *f*′(*η*) of the magnetic field *M* is seen in Fig 8. The comparison of curves with the same *n* (say *n* = 1.4) indicates that the velocity boundary-layer thickness diminishes with snowballing values of *M*. This means that the rise in magnetic field strength boosts the nanofluid flowing beside the boundary-layer. The Lorentz force generated in that case by applying the magnetic transverse field counters viscous forces and reduces movement of the nanofluid [52]. Fig 9 presents the temperature outlines for diverse amounts of magnetic field *M* under slip conditions. With the magnetic field *M* upsurges, the energy of power law nanoliquid declines for an assumed remoteness from the flatness porous surface. This is a result of the magnetic transverse field raises the fluid speed inside the boundary layer, rising the transfer rate, and lowering the thickness of the thermal boundary layer.

### 5.5. Effect of solid fraction *ϕ*

**Fig 10:**
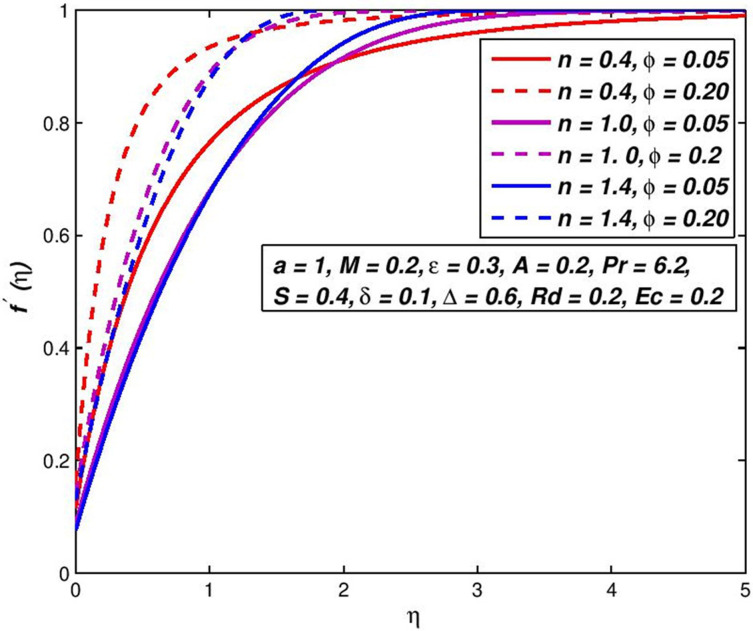
Effect of *ϕ* on *f*′(*η*) for diverse *n* values.

**Fig 11:**
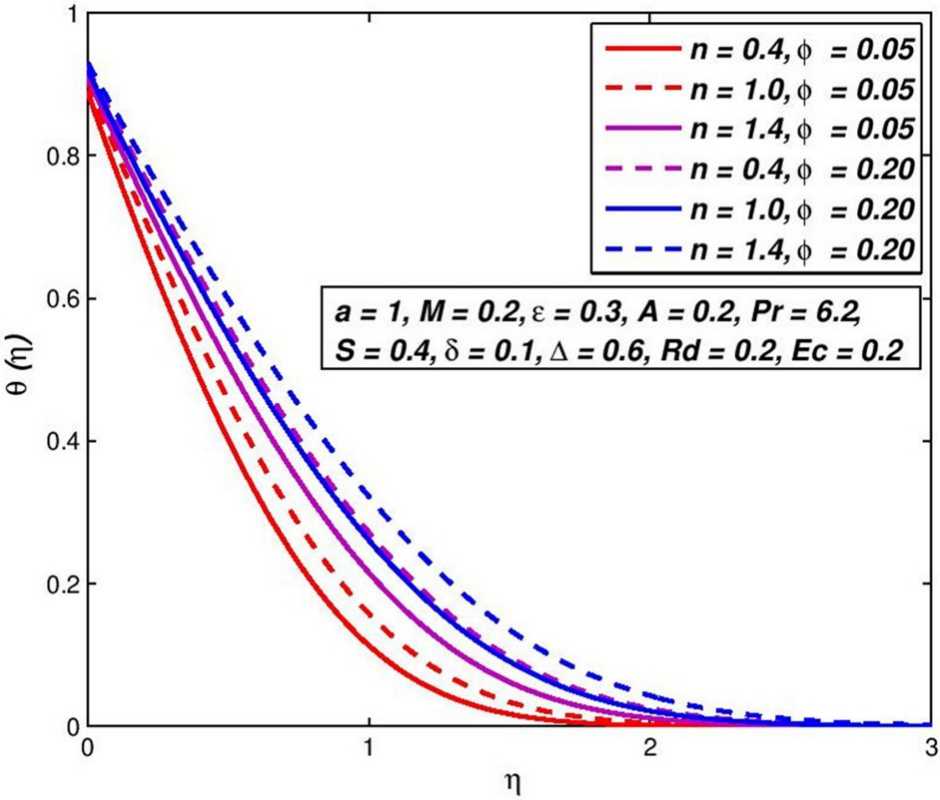
Effect of *ϕ* on *θ*(*η*) for diverse *n* values.

The existence of non-Newtonian nanofluid, the distribution of velocity and temperature, respectively shows in Figs 10 and 11 for the shift in the solid fraction of the nanoparticles volume *ϕ*. It is evident that *f*′(*η*) and *θ*(*η*) of upsurge with the incrementation values of *ϕ*. Physically, these results accord with the performance of dissipating the boundary of impulse and consequently reducing temperature transfer in the boundary layer due to the denser section of nanoparticle’s volume.

The increase in nanoparticle volume increases the total thermal conductivity of nanofluids as the solid particles have larger thermal conductance than standard liquid, which leads to a decrease in the thickening of impulse limits and an increase in thickness of thermal boundaries.

### 5.6. Effect of slip velocity *δ*

**Fig 12:**
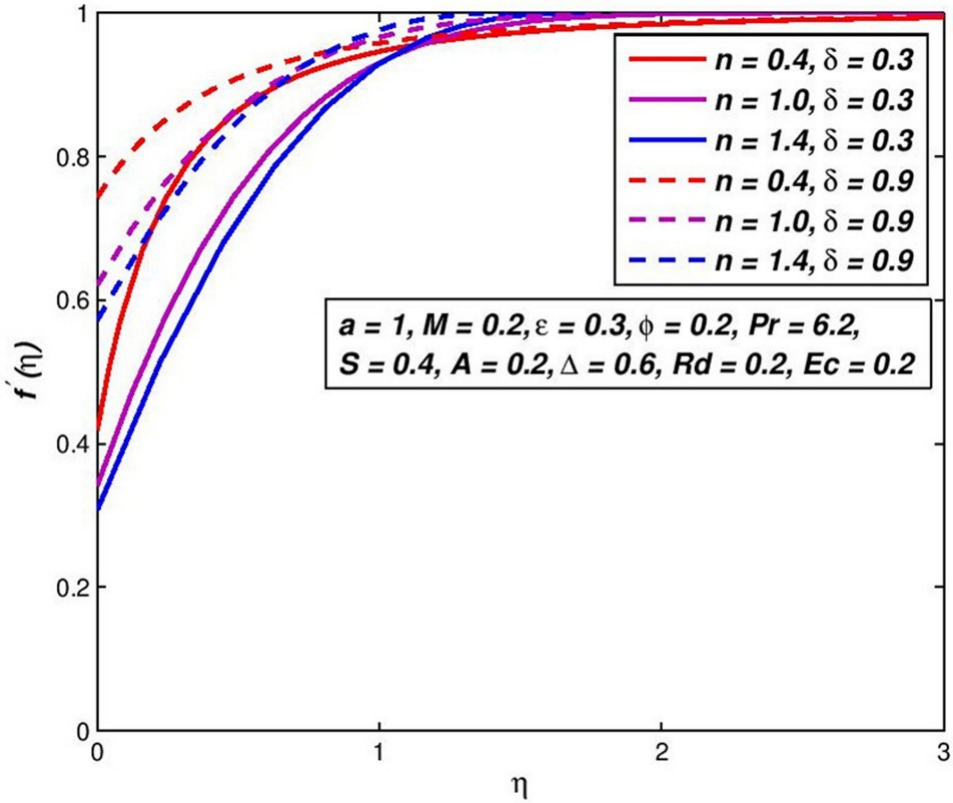
Effect of *δ* on *f*′(*η*) for diverse *n* values.

**Fig 13:**
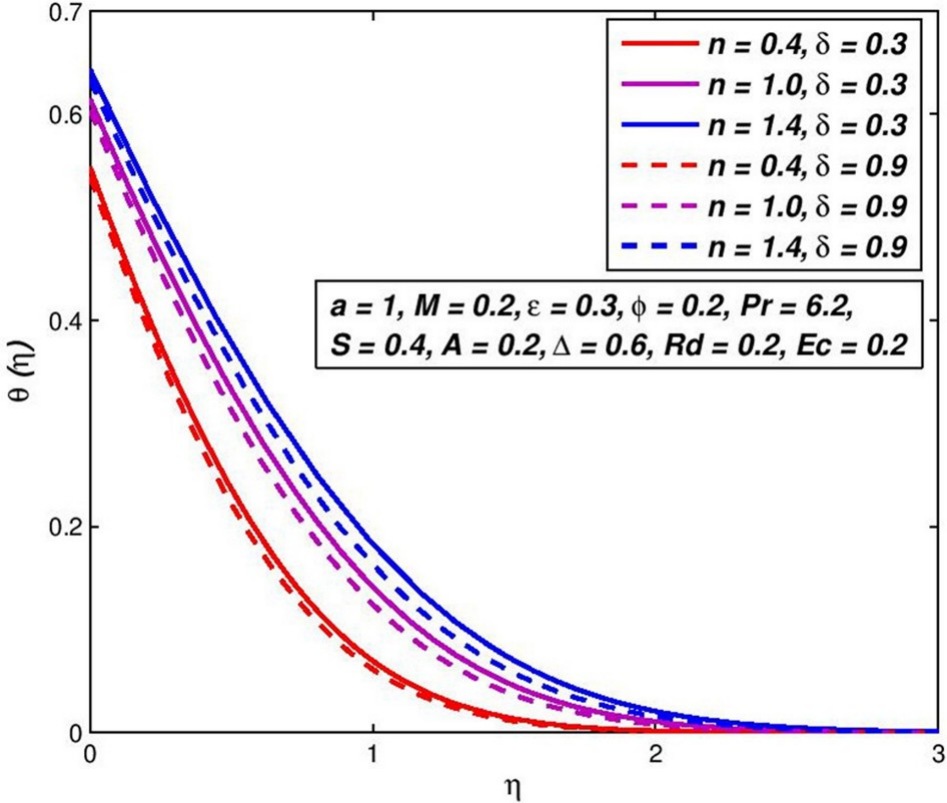
Effect of *δ* on *θ*(*η*) for diverse *n* values.

The impact of change in slip velocity parameter *δ* on nanofluid velocity *f*′(*η*) is shown in Fig 12. It can be seen the pseudoplastic velocity, Newtonian and dilatant nanofluids all boost with growing amounts of slip velocity at the boundaries. This predictable behavior results in non-negative amounts of fluid swiftness at the borderline and consequently the boundary-layer thickener declines with an upsurge in *δ*. Observe that the swiftness of pseudoplastic nanofluids growths speedy tracked by Newtonian and then dilatant nanofluids. On the other side, the surface slippage of the fluid is apparent from Fig 13, which inverts the fluid temperature; i.e., a slip-parameter rise helps to decline the non-Newtonian nanofluid temperature and to raise heat transfer speeds. This results that, due in the increase in the sliding velocity, which increases the collisions between the inner particles of the nanofluid, which leads to an increase in the fluid velocity.

### 5.7. Effect of injection (Suction) *S*

**Fig 14:**
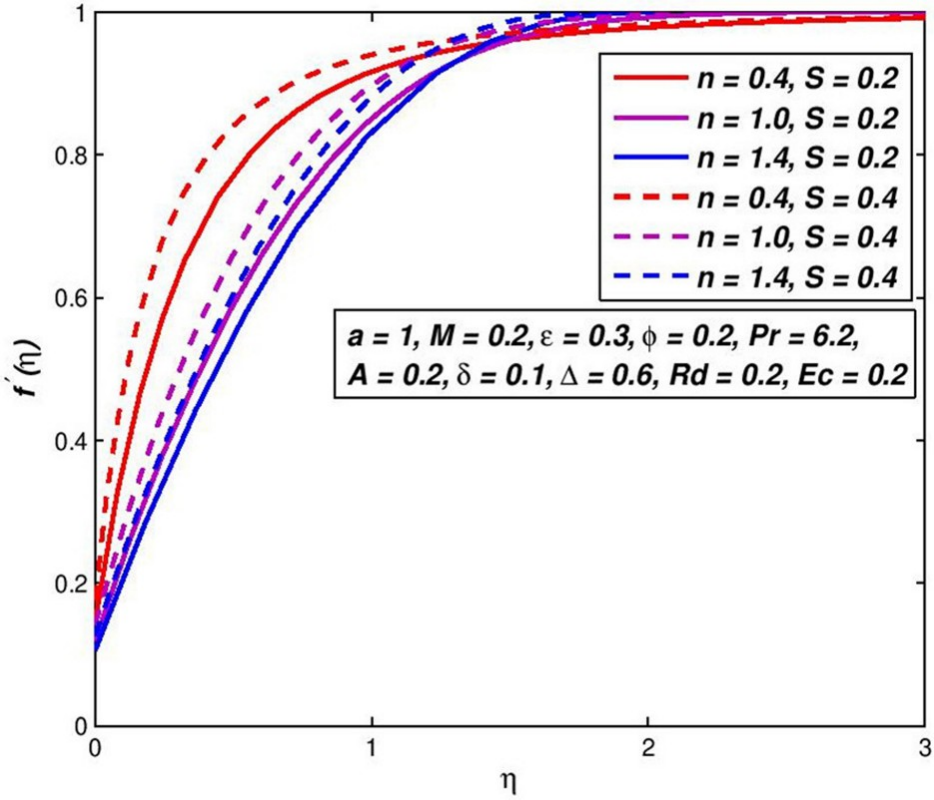
Effect of *S* > 0 on *f*′(*η*) for diverse *n* values.

**Fig 15:**
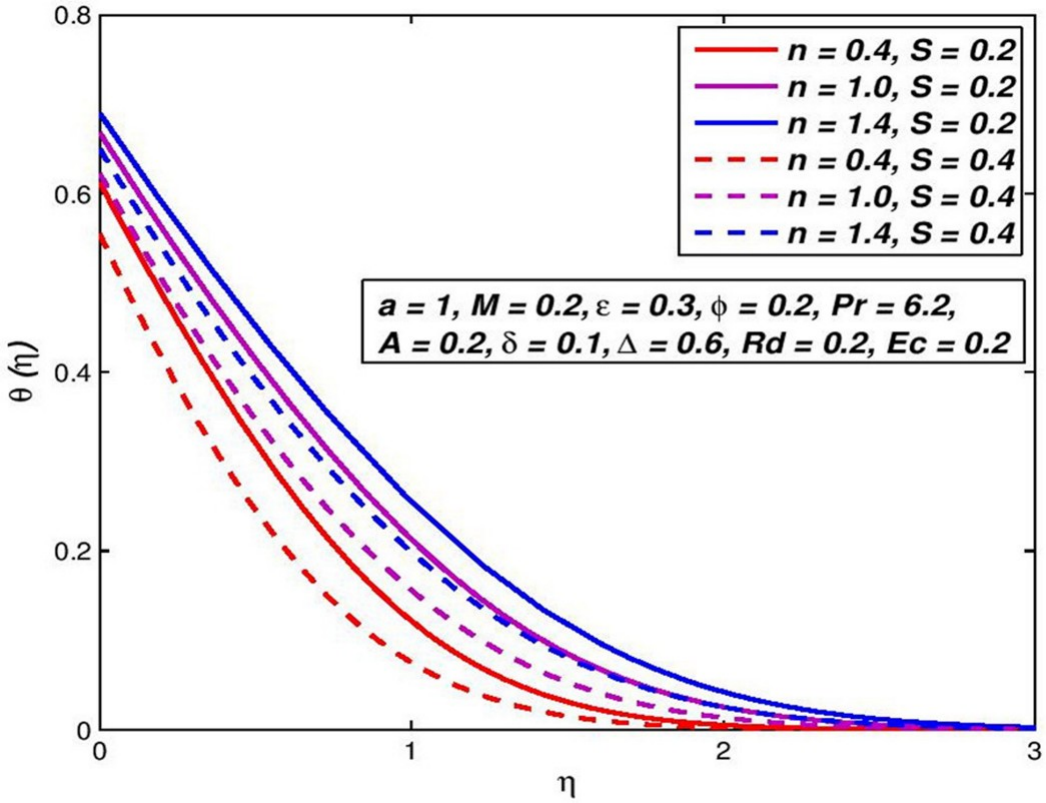
Effect of *S* > 0 on *θ*(*η*) for diverse *n* values.

**Fig 16:**
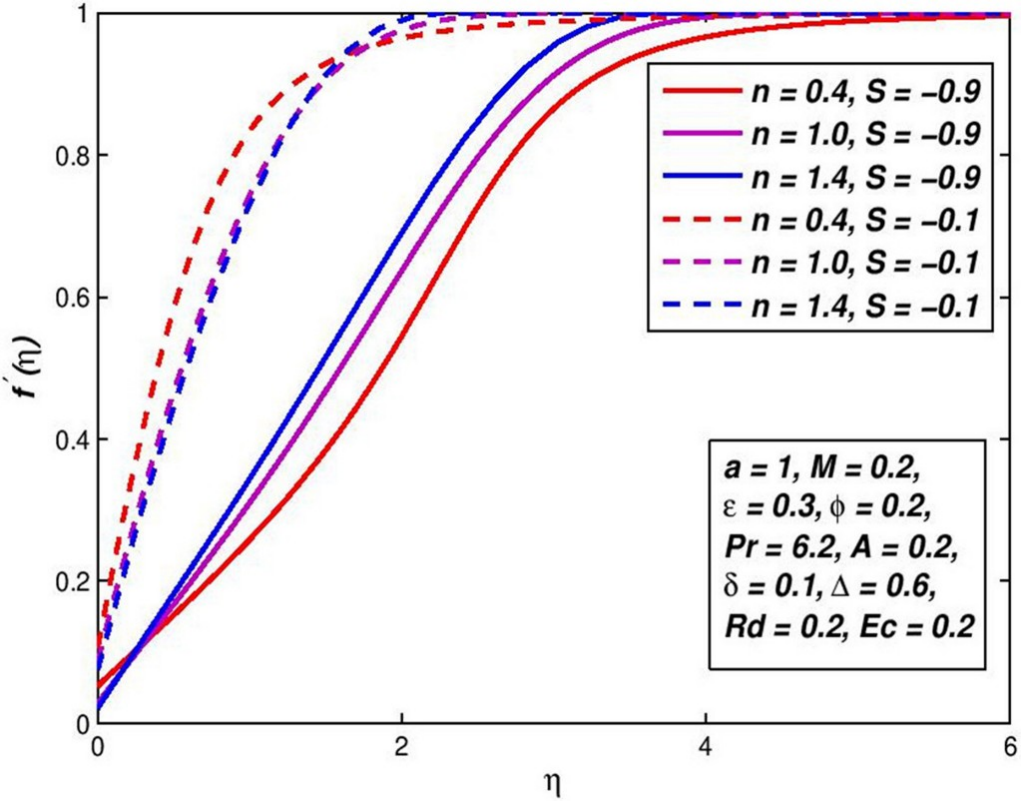
Effect of *S* < 0 on *f*′(*η*) for diverse *n* values.

**Fig 17:**
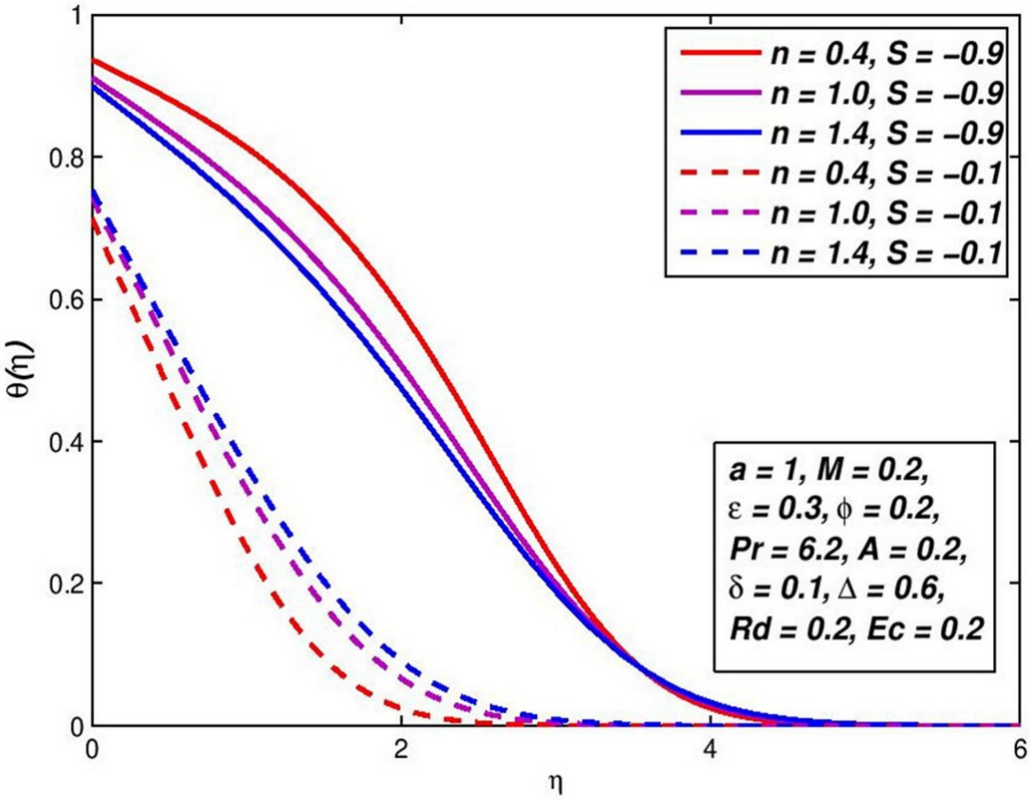
Effect of *S* < 0 on *θ*(*η*) for diverse *n* values.

The effect of variation in suction/injection parameter *S* on the *f*′(*η*) and *θ*(*η*) outlines of H_2_O based nanoliquid are seen in Figs 14–17, respectively. The findings show that *S* > 0 has contributed to a fluid velocity rise, as more fluid is sucked into the pore wall and the thickening of the boundary movement layer is decreased. Conflicting attitude is detected for *S* < 0. In the status of thermal distribution across the border layer, suction on the surface allows the thermal boundary layer thickness to be decreased and the thickener of the thermal bounder layer to be raised through injection. This is due to the porous holes in the surface which are a very important factor in controlling the speed and temperature of the mixture.

### 5.8. Effect of thermal slip Δ

**Fig 18:**
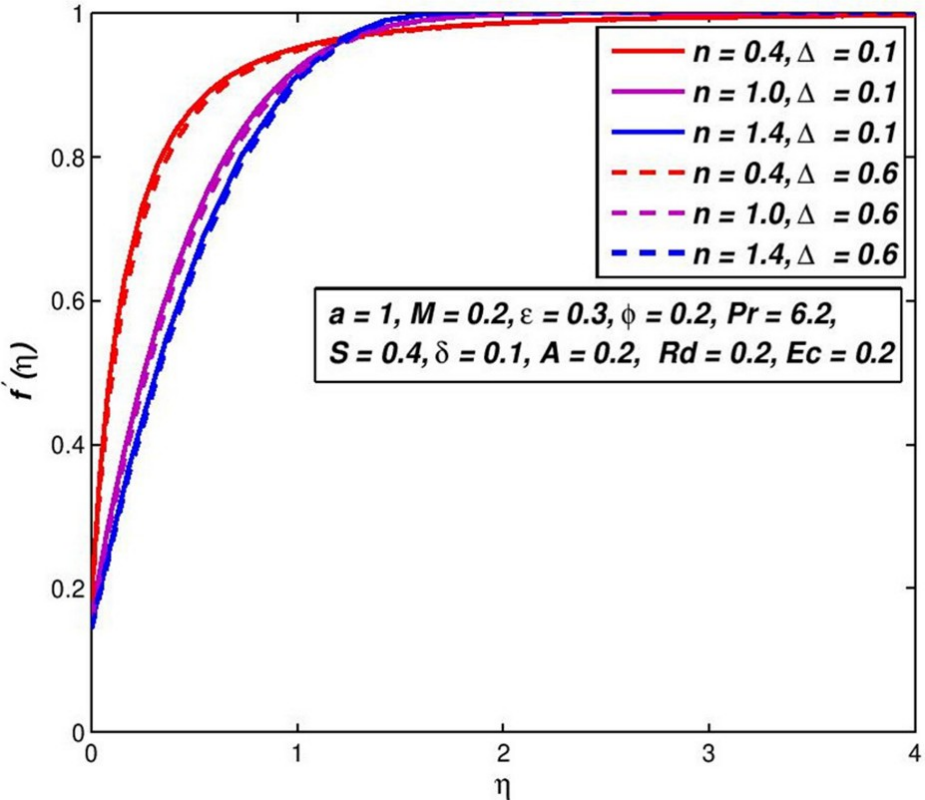
Effect of Δ on *f*′(*η*) for diverse *n* values.

**Fig 19:**
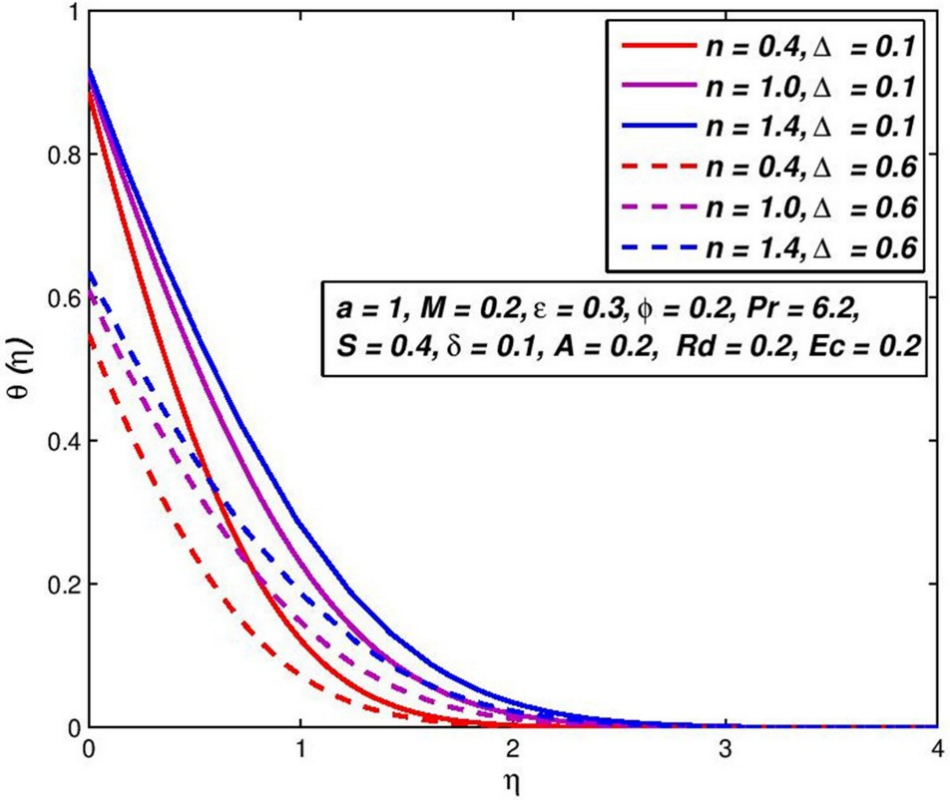
Effect of Δ on *θ*(*η*) for diverse *n* values.

The changes on *f*′(*η*) and *θ*(*η*) outlines with Δ parameter are presented in Figs 18 and 19. If the thermal slip Δ increases, for a certain distance from a flat porous surface a fluid temperature *θ*(*η*) is decreased. Owing to the fluid on the flat porous plate with a temperature lower than the flat porous plate, the thickness of the thermal boundary layer reduces.

### 5.9. Effect of Prandtl number *Pr*

**Fig 20:**
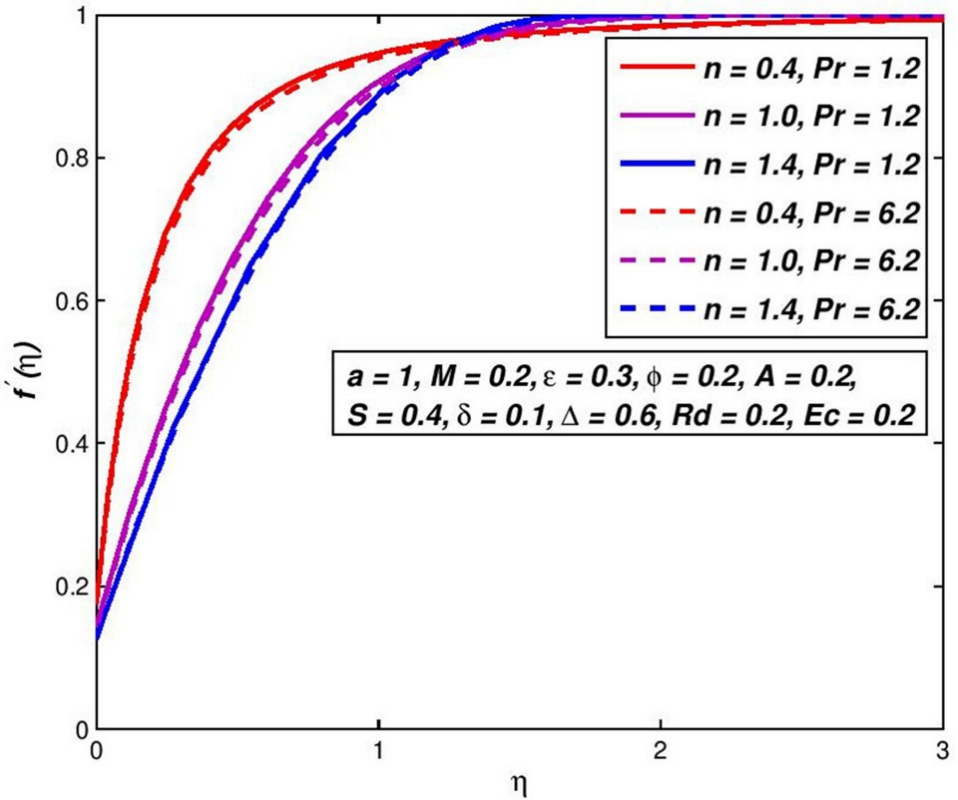
Effect of *Pr* on *f*′(*η*) for diverse *n* values.

**Fig 21:**
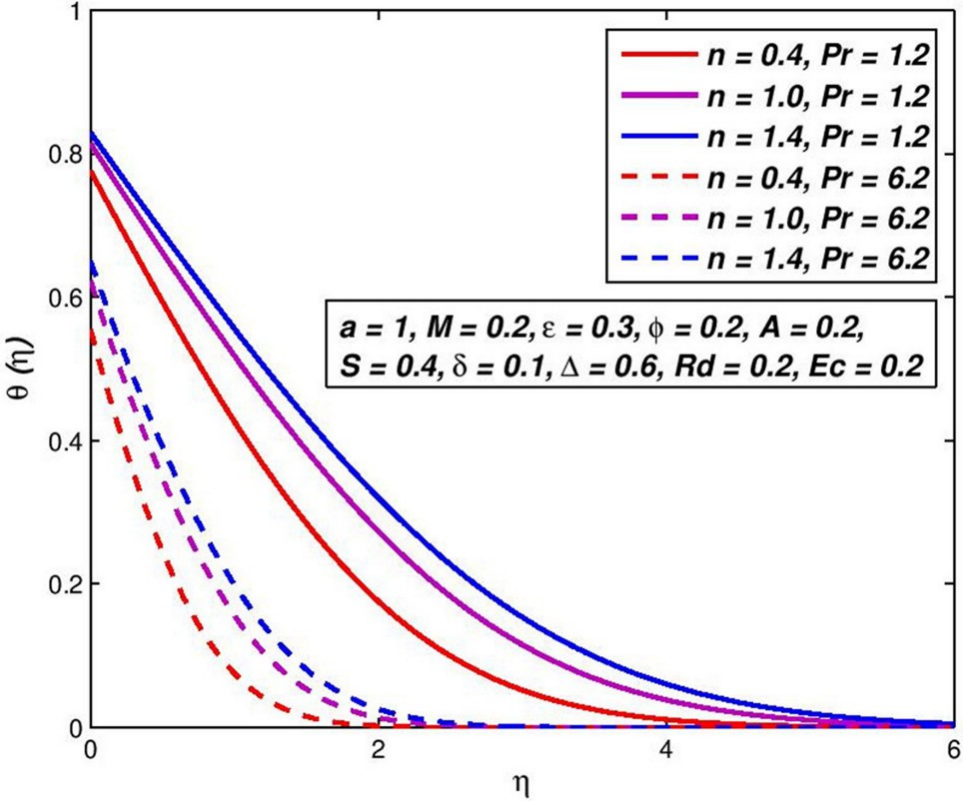
Effect of *Pr* on *θ*(*η*) for diverse *n* values.

Prandtl number is specified as the proportion of motion diffusion to thermal diffusion. Due to temperature dependent viscosity, the Prandtl number will also be the velocity profile. Figs 20 and 21 demonstrate the difference in *Pr* and the impact it may have on the velocity and the outline of temperature. As the amount of Prandtl upsurges, with one distance from the flat plate, the temperature of a nanofluid is declines. Table 4 includes a ratio of the convective to conductive heat transmission on the flat porous plate to the usual amount of the flat porous plate and on its surface, under a variety of different conditions. The pseudoplastic nanofluids at the surface are the maximum nanofluid temperature *θ*(*η*), followed by the Newtonian and dilatant nanofluids which diminish beside the surface. This is compliant with the observed growth of Nusselt when *n* is reduced when all other parameters are kept constant. A rise in *M* or a reduction in *Pr* corresponds to an increment in the transport rate of heat. Increased slip velocity *δ* or slip temperature Δ parameters have resulted in a decrease in the heat transport rate. The temperature of the fluid on the permeable plate is similar to the temperature of the bulk fluid and the Nusselt number is near zero so the velocity or slip parameter is increased into a limitless slip state. The previous debate indicates that an increase in the amount of Nusselt is equivalent to an increase in thermal propagation and the thinning of the thermal boundary-layer.

**Table 4:** Values of $C_{f}Re_{x}^{1/2}$ and $Nu_{x}Re_{x}^{-1/2}$ for diverse flowing parameters.

*n*	*A*	*ϕ*	*ε*	*M*	*P*_*r*_	*S*	*δ*	Δ	*Rd*	*Ec*	_ $C_{f}Re_{x}^{1/2}$ _	$Nu_{x}Re_{x}^{-1/2}$
0.4	0.2	0.20	0.3	0.2	0.7	0.2	0.1	0.6	0.2	0.2	1.758611	0.039423
	0.6										1.672878	0.039196
	1.2										1.580010	0.038923
1.0	0.2										1.424609	0.629927
	0.6										1.341474	0.626597
	1.2										1.243112	0.622713
1.4	0.2										1.268867	0.252270
	0.6										1.189394	0.251147
	1.2										1.093572	0.249820
0.4		0.05									1.117823	0.044398
		0.10									1.300255	0.042610
		0.20									1.758302	0.039422
1.0		0.05									0.786755	0.721288
		0.10									0.968909	0.687798
		0.20									1.424611	0.629927
1.4		0.05									0.672798	0.290187
		0.10									0.830456	0.276527
		0.20									1.268860	0.252345
0.4			0.0								1.460863	0.036462
			0.1								1.461721	0.035729
			1.6								1.465353	0.032438
1.0			0.0								1.191304	0.594142
			0.1								1.192264	0.579897
			1.6								1.196248	0.518059
1.4			0.0								1.059521	0.240951
			0.1								1.060471	0.234802
			1.6								1.064390	0.208475
0.4				0.2							1.463302	0.034339
				0.6							1.637041	0.912670
				0.9							1.735443	0.036615
1.0				0.2							1.194012	0.553349
				0.6							1.505156	0.600293
				0.9							1.683143	0.624331
1.4				0.2							1.062236	0.223427
				0.6							1.417140	0.246055
				0.9							1.621579	0.257743
0.4					0.7						1.793756	0.015556
					1.2						1.788058	0.019834
					6.2						1.758611	0.039423
1.0					0.7						1.455183	0.243399
					1.2						1.450146	0.309846
0.4					0.7						1.793756	0.015556
					1.2						1.788058	0.019834
					6.2						1.424609	0.629927
1.4					0.7						1.297349	0.096722
					1.2						1.292728	0.122978
					6.2						1.268867	0.252271
0.4						-0.9					0.524780	0.005622
						-0.1					1.056315	0.025423
						0.0					1.183704	0.028549
						0.2					1.463302	0.034339
						0.4					1.758611	0.039423
1.0						-0.9					0.281256	0.147334
						-0.1					0.859250	0.430053
						0.0					0.967947	0.471922
						0.2					1.194012	0.553349
						0.4					1.424609	0.629927
1.4						-0.9					0.211778	0.072482
						-0.1					0.759836	0.178117
						0.0					0.858514	0.193381
						0.2					1.062236	0.223429
						0.4					1.268867	0.252277
0.4							0.0				1.991399	0.039111
							0.3				1.398453	0.039922
							0.9				0.826174	0.040777
1.0							0.0				1.608440	0.621801
							0.3				1.139310	0.642189
							0.9				0.690132	0.660807
1.4							0.0				1.425028	0.249429
							0.3				1.025536	0.256855
							0.9				0.636041	0.264341
0.4								0.0			1.929800	0.649348
								0.1			1.904636	0.638665
								0.6			1.834660	0.597380
1.0								0.0			1.650664	0.976440
								0.1			1.631676	0.903696
								0.6			1.572022	0.650476
1.4								0.0			1.511053	0.377645
								0.1			1.494633	0.352785
								0.6			1.440644	0.262693
0.4									0.0		1.754550	0.042530
									0.2		1.758611	0.039423
									0.6		1.765694	0.034996
1.0									0.0		1.420119	0.683151
									0.2		1.424609	0.629927
									0.6		1.430725	0.555668
1.4									0.0		1.264742	0.273982
									0.2		1.268867	0.252271
									0.6		1.274466	0.222117
0.4										0.0	1.753971	0.042277
										0.6	1.759045	0.039209
										0.9	1.769704	0.032446
1.0										0.0	1.421264	0.671605
										0.6	1.425569	0.620199
										0.9	1.434555	0.509579
1.4										0.0	1.266071	0.267851
										0.6	1.269990	0.247118
										0.9	1.278226	0.202688

### 5.10. Effect of radiation parameter *Rd*

**Fig 22:**
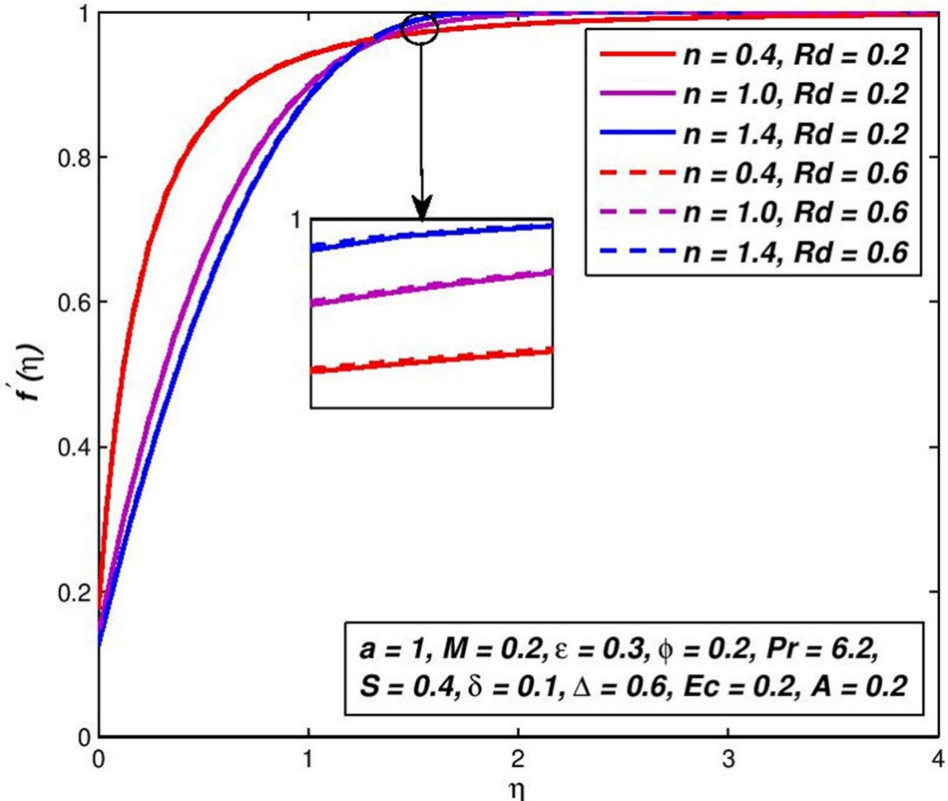
Effect of *Rd* on *f*′(*η*) for diverse *n* values.

**Fig 23:**
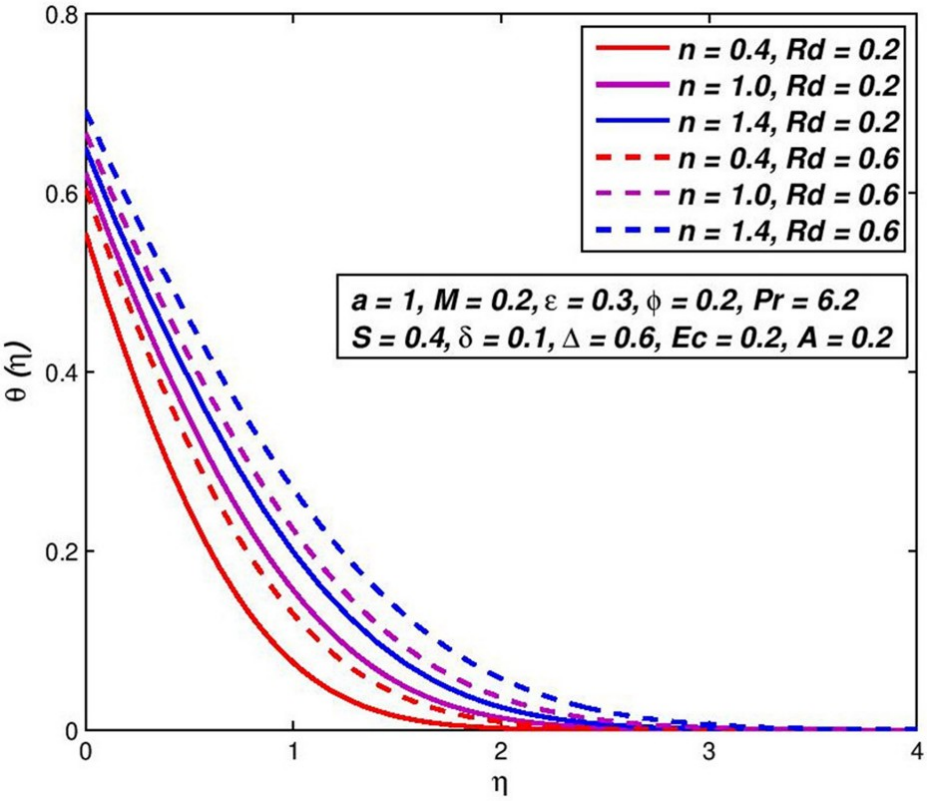
Effect of *Rd* on *θ*(*η*) for diverse *n* values.

Figs 22 and 23 visualize the primary *f*′(*η*) velocity and temperature *θ*(*η*) with the change in radiative flow parameter *Rd*. Heat radiation in different manufacturing systems is a physical heat transfer mode, which implies new thermal applications. It rises in the amplified thermal diffusivity bound, $\left(1+\epsilon \theta +\frac{4}{3}Rd\right)\theta^{\prime \prime }$ in the energy preservation formula (3.8). The inclusion of the denominator of the term thermal radiative flux demotivates the magnetic fluid regime and greatly depletes the main velocity (Fig 22) and even temperatures (Fig 23) for both dilatants and pseudo-plastic liquids. For pseudo-plastic and dilatory nanofluids the thickness of the velocity and temperature boundary-layer are decreased, while thicker velocity and temperature boundary-layers for thinning polymers are generated (pseudo-plastic).

### 5.11. Effect of Eckert number *Ec*

**Fig 24:**
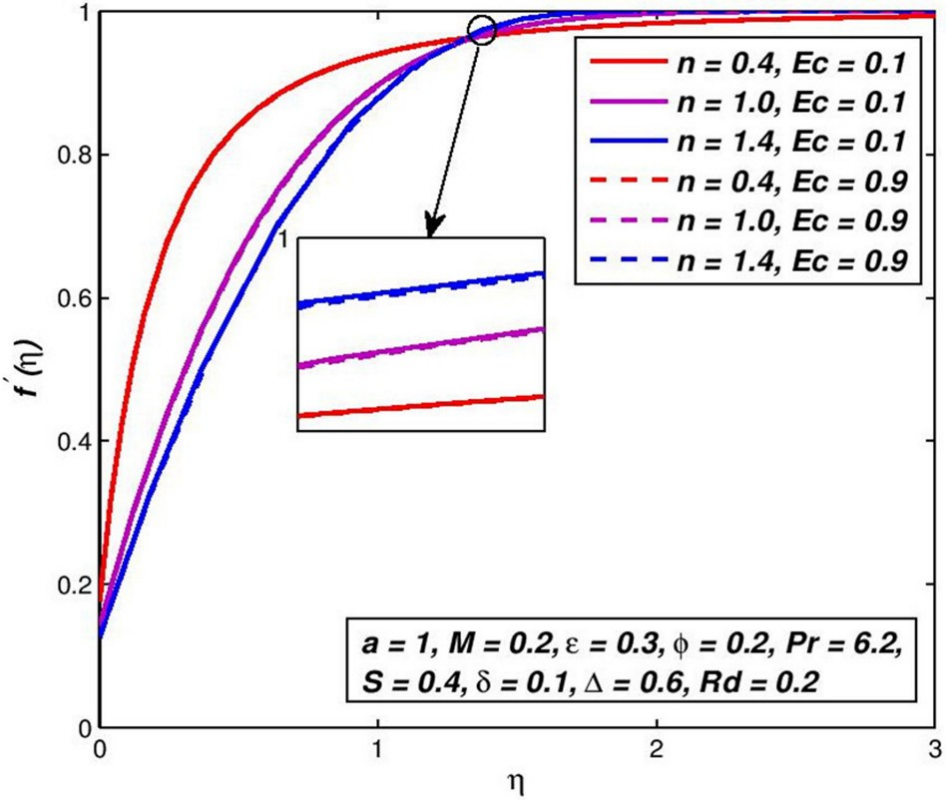
Effect of *Ec* on *f*′(*η*) for diverse *n* values.

**Fig 25:**
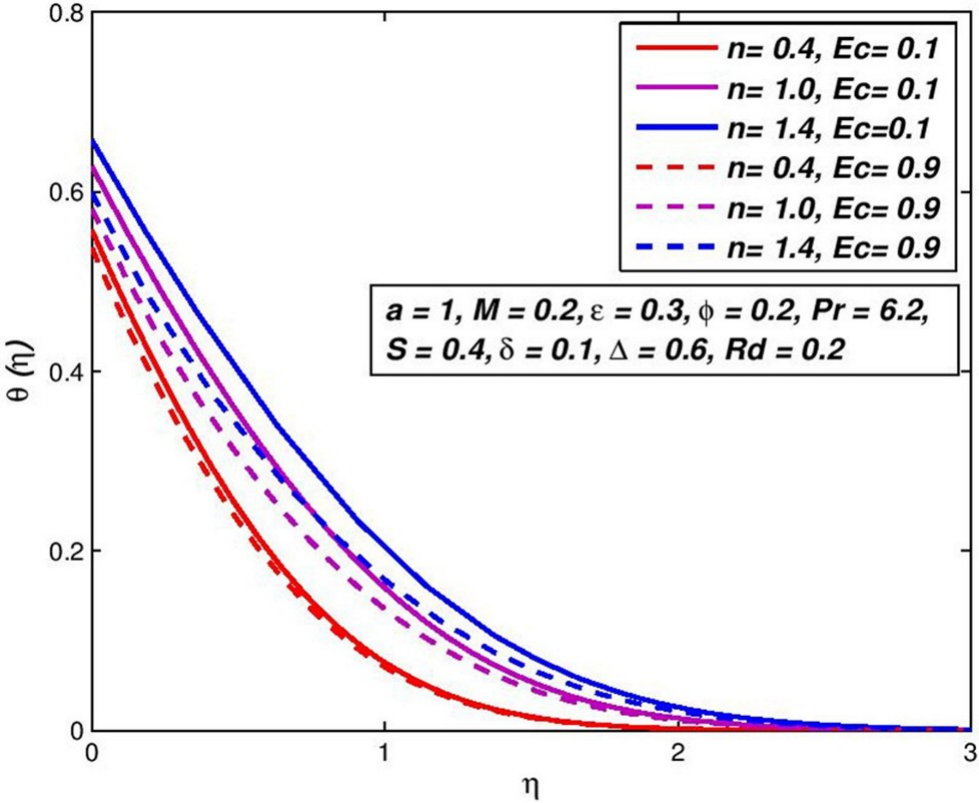
Effect of *Ec* on *θ*(*η*) for diverse *n* values.

Figs 24 and 25 show the response in primary *f*′(*η*) velocity and temperature *θ*(*η*) with the change in *Ec* for both the states. In the field of continuum mechanics, *Ec* has an important function. This non-dimensional number will successfully connect the relationship between the variations in boundary layer enthalpy and the kinetic energy. *Ec* values lead to a substantial rise in the main velocity, and more of the pseudo-plastic status increases with the same *Ec* increase. Overall, though the maximum primary velocity is related to the expansion case as before (see Fig 24).

Temperatures are also strongly enhanced for Ec, and the thickener of the temperature boundary-layer is high; the enhancement for pseudoplastic is greater than that of dilatants polymer. Again, it is worth noting that a lack of this terminology contributes to a large projected temperature in many thermo-magneto flow simulations (and a simultaneous over-predicted number of Nusselt).

### 5.12. Flow factors influences on drag force and heat transport rate

In Table 4, the influences of the flux-controlled parameters on the drag force factor (*C*_*f*_) and local Nusselt number (*Nu*_*x*_) are acquired.

## 6. Conclusion and future study

The key effects on the slips and heat transfer properties of non-Newtonian power-law nanofluid through a porous, smooth surface of variable viscosity, variable thermal conductivity, and applied transverse magnetic fields were evaluated numerically during this work. No such research is described in the literature to the best of the authors’ understanding. In the bounds of shear-stress, viscosity and thermal conductance are known as a linearity function of temperature and slip constraints. The transformed set of equations are solved utilizing Keller-box technique. Diagrams and tables display numerical equations for the velocity and temperature change of the Cu-H_2_O nanofluid beside the boundary-layer. The variance results in various controlling parameters are discussed in the prior section on velocity and temperature distributions. It is concluded:

1. The pseudoplastic nanofluids have the largest worth of a drag force factor to keep track of the Newtonian nanofluids and then the dilatant nanofluids. This is results in the boost rate in fluid velocity is highest for shear thinning fluids due to the lowest rate of effective viscosity at the surface. The incrementation heat transport rate is detected for *S* < 1 and the lowermost for the status of *S* > 1.
2. The increase in viscosity of nanofluid and nanoparticles reduces speed and raises the nanofluid temperature within the border layer. This decreases the rate of heat transportation and boosts the boundary-layer thickener of the momentum.
3. The intensity improvement of the utilized magnetic transverse field and suction parameter boosts nanofluid velocity and reduces the temperature inside the boundary-layer.
4. The upsurge in both slip factors diminishes boundary-layer thickness of the momentum. Although the rise in the velocity slippy improves the rate of heat transmit and the rate of heat transmit is decreased with thermal slippy.

## Abbreviations

*a*, *b*: constants
*A*: viscosity parameter
*B*_0_: uniform magnetic field
*C*_*f*_: skin friction coefficient
*Ec*: Eckert number
*T*: temperature
*T*_*w*_: temperature at surface
*T*_*∞*_: ambient temperature
*u*_*∞*_: ambient velocity
*V*_*w*_: velocity of injection (suction)
(*x*,*y*): Cartesian coordinate system
*u*,*v*: velocity components
*C*_*p*_: specific heat
*q*_*r*_: flux of radiative heat
*L*: preliminary velocity slip
*L*_1_: velocity slip
*D*: preliminary thermal slip
*D*_1_: thermal slip
*f*: dimensionless stream function
*Rd*: thermal radiation
*R*_*e*_: Reynolds number
*k**: mass absorption coefficient
*M*: magnetic parameter
*m*: shape factor
*Nu*: local Nusselt number
*n*: power-index
*Pr*: Prandtl number
*q*_*w*_: wall heat flux
*S*: injection (suction) parameter
*Pr*: Prandtl number
***Greek symbols***
*ρ*: density
*μ*: dynamic viscosity
*ε*: thermal conductance parameter
*v*: kinematic viscosity
*κ*: thermal conductivity
*σ*: electrical conductance
*δ*: velocity slip
*Δ*: thermal slip
*ψ*: stream function
*η*: similarity variable
*θ*: dimensionless temperature
*σ**: Stephan-Boltzmann constant
*ϕ*: volume fraction
***Subscripts***
*f*: base liquid
*s*: solid nanoparticles
*n*_*f*_: hybrid nanofluid, nanofluid
